# The *p53* mRNA exhibits riboswitch-like features under DNA damage conditions

**DOI:** 10.1016/j.isci.2025.113555

**Published:** 2025-09-12

**Authors:** Sa Chen, Lixiao Wang, Laurence Malbert-Colas, Konstantinos Karakostis, Vanesa Olivares-Illana, Sivakumar Vadivel Gnanasundram, Robin Fahraeus

**Affiliations:** 1 Department of Medical Biosciences, Umeå University, Building 6M, 901 85 Umeå, Sweden; 2 Inserm UMRS 1342, Institut de Recherche St Louis, Université Paris Cité, 75010 Paris, France; 3 Biochemistry and Molecular Biology Department, University of Valencia, Valencia, Spain; 4 Laboratorio de Interacciones Biomoleculares y Cáncer. Instituto de Física Universidad Autónoma de San Luis Potosí, Manuel Nava 6, Zona Universitaria, San Luis Potosí 78290, SLP, México; 5 Department of Biological Sciences & Engineering, Indian Institute of Technology Palakkad, Palakkad 678 623, India; 6 RECAMO, Masaryk Memorial Cancer Institute, Zluty kopec 7, 65653 Brno, Czech Republic

**Keywords:** Molecular biology, Structural biology

## Abstract

RNA riboswitch structures control prokaryotic gene expression in response to changes in the cellular environment, but how this concept has evolved in mammalian cells is yet little known. Here, we describe the riboswitch-like features of the *p53* mRNA that controls p53 synthesis following DNA damage. The conserved BOX-I stem-loop in the 5′ coding sequence acts as an aptamer that controls the folding of a compact downstream MDM2-binding *p53* mRNA structure. MDM2 brings the *p53* mRNA to the ribosome and promotes p53 synthesis. High-throughput in-cell RNA structural probing and *in vitro* RNA-RNA and RNA-protein interactions show how the cancer-associated synonymous mutation in codon 22 (CASM22) of the BOX-I aptamer stabilizes the *p53* mRNA structure and prevents the formation of the MDM2-binding platform. However, the CASM22 does not affect *p53* mRNA folding during the unfolded protein response, demonstrating the specificity by which the CASM22 targets the p53 DNA damage response.

## Highlights

• A riboswitch-like mRNA structure controls p53 synthesis following DNA damage
• A conserved aptamer structure controls a compact downstream MDM2-binding platform
• The *p53* mRNA-MDM2 interaction is sufficient to induce p53 synthesis
• A cancer-associated synonymous mutation disrupts the folding of the p53 mRNA

## Graphical abstract

**Figure:**
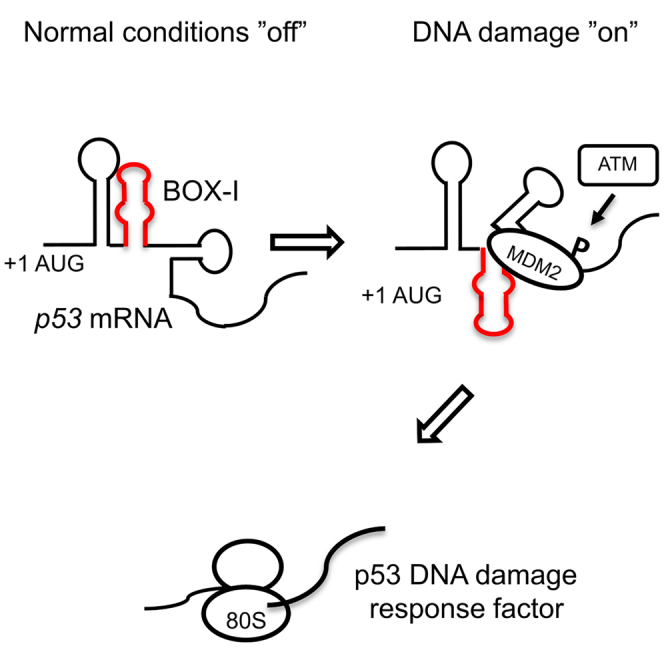

## Introduction

Structures within mRNAs play important roles in mediating the interaction with RNA-binding proteins to control mRNA stability and translation.^1^ Prokaryotic RNA riboswitches regulate the synthesis of the encoded protein in *cis* in response to changes in the cellular environment that typically includes a ligand-binding aptamer domain that forms a downstream translation initiation factor-binding platform.^2^ The fungal thiamine pyrophosphate switch controls mRNA splicing, but how the concept of riboswitches plays a role in controlling translation of specific mRNAs in mammalian cells in response to changes in intra- or extracellular conditions is still poorly understood.^3^

RNA structure-based gene regulation depends on the ability to form dynamic structural ensembles.^4^^,^^5^^,^^6^ Technical advancements to determine RNA structures at high resolutions such as nuclear magnetic resonance and single-particle cryo-electron microscopy (cryo-EM) coupled with advanced computational modeling tools have given new insights into RNA structures.^7^ The development of highly reproducible *in vivo* techniques holds promise to determine changes in RNA structures and RNA structure-based functions in response to signaling pathways.^8^ It is highly anticipated that this will provide a more comprehensive picture of how RNA structures are integrated in the control of gene expression and their roles in the development of disease.

The p53 tumor suppressor is mutated in more than 50% of human cancers and plays a pivotal role in the cellular response to various changes in the cellular environment, including DNA damage, by controlling the expression of downstream target genes.^9^ MDM2 is a key regulator of p53 that under normal conditions binds the highly conserved BOX-I-encoded peptide located in the p53 N terminus and targets p53 for degradation.^10^ However, during DNA damage, MDM2 and its homolog MDMX are activated by the ATM kinase via phosphorylation on serine 395 and 403, respectively. MDM2 promotes the expression of the full-length p53 protein to restore genomic integrity. MDMX acts as an RNA chaperone on the nascent *p53* mRNA to form an MDM2-binding platform by releasing the highly conserved stem-loop II, known as BOX-I, from stem-loop I. This includes disrupting the interaction between codon 10 in stem-loop I and codon 21 in stem loop II.^11^^,^^12^^,^^13^^,^^14^

The *p53* mRNA also plays a role in controlling the functional diversities of p53 in response to different stress signaling pathways.^15^^,^^16^^,^^17^ For example, activation of the PERK kinase^18^ during the unfolded protein response (UPR) results in a *p53* mRNA conformation that promotes translation initiation from the second in-frame AUG at +118 and the expression of the p53/47 isoform.^19^^,^^20^

Synonymous mutations (SMs) are overrepresented in cancers and linked to poor prognosis, implicating that RNA sequences affect the encoded proteins.^21^^,^^22^^,^^23^ Previous works have shown that the cancer-associated SM in T*p53* at codon 22 (CASM22) prevents p53 activation during the DNA damage response (DDR),^11^ but little is still known how single SMs affect RNA three-dimensional (3D) structures and how this affects the activity of the encoded protein.

Here, we have used high-throughput in-cell RNA structure probing and computational modeling tools together with biochemical and cell biological assays to illustrate the riboswitch-like features of the *p53* mRNA in response to DNA damage. The highly conserved BOX-I stem-loop within the first 120 nucleotides (nts) of the *p53* mRNA coding sequence acts as an aptamer that shapes the folding of a compact downstream MDM2-binding platform during the DDR to promote p53 synthesis. The CASM22 is located within the BOX-I and prevents the folding of the MDM2-binding platform.

## Results

### A conserved stem-loop structure within the *p53* mRNA coding sequence controls a compact downstream MDM2-binding platform and p53 expression

**Figure 1:**
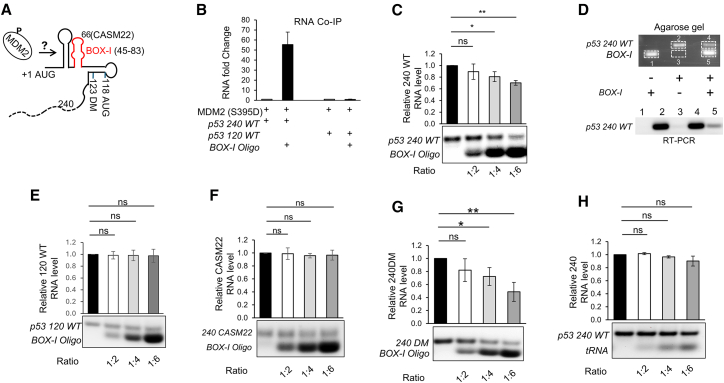
The highly conserved *p53 BOX*-I stem-loop promotes a downstream compact MDM2-binding platform (A) A diagram illustrating the first 240 nts of the *p53* coding sequences. The highly conserved BOX-1 stem-loop is indicated in red, and the positions of the cancer-associated synonymous mutation at position +66 in codon 22 (CASM22) and the double synonymous mutation (DM) are indicated, as well as the second in-frame AUG at +118^13^ (see also Figure S1). (B) RNA coIP using anti-MDM2 antibodies followed by RT-qPCR shows the relative amount of 1 pmol indicated *p53* RNAs (+1 to +240 or +1 to +120 nts) bound to MDM2(S395D) (40 pmol) following addition of 6 pmol free *BOX-I* RNA oligonucleotides (+45 to +83 nts) (see also Figure S2). (C) Gel shift assays show the migration of 240 *p53* WT RNA (1 pmol) following the addition of free *BOX-I* RNA oligonucleotides (2, 4, and 6 pmol) (see also Data S1). (D) Like in (C). The gel shift bands (upper panel, numbers 1 to 5) were cut out, and RT-PCR (lower panel) was used to determine the presence of the *p53* 240 WT RNA in each band in the presence of 6 pmol of *BOX-I* RNA. (E) Addition of free *BOX-I* RNA oligonucleotide to the *+*120 *WT* RNA. (F) The migration of the +240 nts *p53* RNA carrying the cancer-associated synonymous mutation in codon 22 (CASM22) in the presence of increasing amounts of the *BOX-I* RNA oligonucleotide. (G) Migration of the +240 *CASM22* RNA with an additional synonymous mutation in codon 41 (double mutant; DM). (H) Migration of the +240 *p53* WT RNA in the presence of increasing amounts of tRNA. See also Figure S2. The data represent the average of at least three independent experiments. ∗*p* < 0.05, ∗∗*p* < 0.01.

We were interested to understand ATM kinase-induced structural changes in the *p53* mRNA that allows the MDM2-*p53* mRNA interaction to take place in response to DNA damage and the consequent induction of p53 expression. It was previously shown that the sequence between the two in-frame AUGs (+1 and +118) of the *p53* mRNA is required for MDM2 interaction, and it was proposed that MDM2 following phosphorylation on serine 395 binds the highly conserved BOX-I stem-loop (+45 to +83) (Figures 1A and S1). However, RNA coimmunoprecipitation (coIP) using anti-MDM2 antibodies showed that the addition of the free *BOX-I* stem-loop RNA oligonucleotide to a *p53* mRNA (+1 to +240 nts) resulted in an approximately 60-fold increase in the interaction between MDM2 carrying the ATM phosphomimetic mutation at serine 395 (MDM2(S395D)). This increase in affinity did not take place when we instead used a *p53* mRNA sequence including the first 120 nts only (+1 to +120 nts) (Figure 1B). This indicates that MDM2 does not interact with the BOX-I stem-loop and that adding free *BOX-I* RNA oligonucleotide acts in *trans* to induce an MDM2-friendly *p53* mRNA structure downstream of +120.

**Figure 2:**
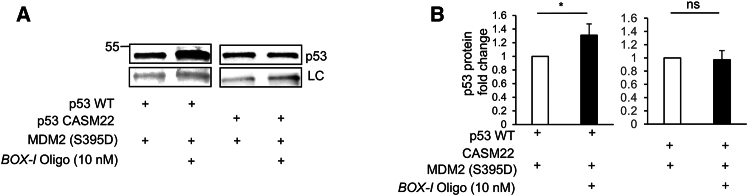
The BOX-I oligo induced p53 expression (A) Western blot showing induction of p53 expressed from the wild-type mRNA, but not the mRNA carrying the CASM22, following addition of 10 nM *BOX-I* oligo for 10 h in the presence of proteasome inhibitor MG132 (10 mM). (B) The graph shows average induction of p53 expression in H1299 cells expressing the indicated p53 construct together with MDM2(S395D) and treated with 10 μM MG132 and with the addition, or not, of 10 nM *BOX-I* RNA oligonucleotides. The western blot shows one representative experiment. LC, loading control. The data represent the average of at least three independent experiments. ∗*p* < 0.05, ns, not significant.

We next made the surprising observation that the +240 nts *p53* RNA migrated faster over a 1.0% agarose gel in the presence of the *BOX-I* RNA oligonucleotide (Figures 1C and 1D; Data S1). This change in migration did not happen in the presence of denaturant formamide, indicating that it is caused by a change in the RNA structure (Figure S2). When the *BOX-I* RNA oligonucleotide was added to the +120 nts *p53* RNA (Figure 1E), or when it was added to a +240 nts *p53* RNA carrying the cancer-associated CASM22 SM (CUA>CUG) at position +66, that is located within the head of the BOX-I stem-loop, it had no effect on the migration of these RNAs (Figures 1A–1F and S1). Previous works have shown that the CASM22 prevents MDM2(S395D) from binding *p53* mRNA and that introducing a compensatory mutation in codon 41 (GAU>GAC) (double mutant [DM]) at position +123 restores MDM2(S395D) binding (Figure S1).^11^^,^^13^ When the *BOX-I* RNA oligonucleotide was added to the +240 nts *p53-DM* RNA, we observed a faster migration, similar to that of the wild-type (WT) RNA (Figure 1G). Increasing amounts of tRNA had no significant effect on the migration of the +240 nts *p53-WT* RNA (Figure 1H). Adding 10 nM *BOX-I* RNA oligonucleotides for 10 h to H1299 cells expressing MDM2(S395D) and WT p53 cDNA resulted in a low, but significant (average 40%), increase in p53 expression (Figures 2 and S3). Importantly, adding *BOX-I* RNA oligonucleotides to cells expressing the *CASM22* RNA did not affect p53 expression levels. These data show the formation of a compact MDM2-binding platform downstream of the first +120 nts of the *p53* mRNA and how the formation of this platform is disrupted by a single cancer-associated mutation in codon 22.

### The CASM22 single-nucleotide mutation stabilizes the *p53* mRNA structure

**Figure 3:**
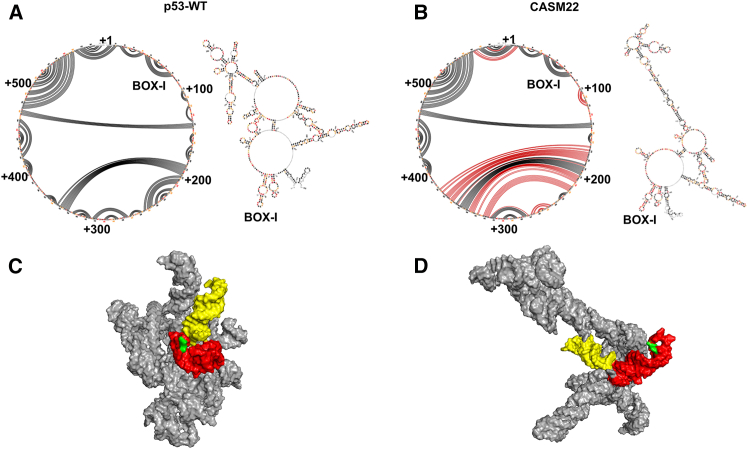
The CASM22 single-nucleotide mutation alters the *p53* mRNA structure Global SHAPE-MaP, secondary structures, and computational 3D modeling analyses of the *p53* mRNA CASM22. The high-confidence base-pairing of *WT* (A) and *CASM22* RNAs (B) are shown in circular plots with their corresponding secondary structures. Altered base-pairing due to CASM22 is shown in red lines, and the position of *BOX-I* stem-loop is indicated. Computational modeling of the 3D structure of *p53* mRNA of WT (C) and CASM22 (D). The first stem-loop is shown in yellow, BOX-I is shown in red, and the position of CASM22 is highlighted in green. See also Figures S5–S7. SHAPE-MaP data shown are representative of at least two independent repeats.

We were interested to know how the BOX-I stem-loop controls the formation of the MDM2-binding platform and how this is prevented by the CASM22. We applied the highly reproducible SHAPE-MaP technique to study changes in large RNA structures *in vivo.*^8^^,^^24^ The global SHAPE-MaP analysis showed that the percentage of nucleotides with median-to-high SHAPE reactivity of *CASM22* is 35.7%, decreased from the high of 40.7% of *p53-WT* (see Data S1). The RNA secondary structures generated from the SuperFold algorithm^24^ based on the SHAPE values showed a gross change in RNA structures spanning sequences downstream of the second AUG at +120. The major alterations in the base-pairing pattern by this single-nucleotide substitution are indicated as red lines in the circular plots (Figures 3A, 3B, and S4). Evaluating the free energy of the CASM22 by using RNAeval from ViennaRNA Package^25^ showed a lower energy as compared to the WT *p53* mRNA (ΔΔG = ΔG_CASM22_-ΔG_p53-WT_ = −2.7 kcal/mol). Although its overall free energy change is not significant, the lower free energy together with the reduced SHAPE reactivity is in line with CASM22 disrupting the dynamics of the *p53* mRNA. Using the computational RNAComposer,^26^^,^^27^ we built a 3D model of the *p53-WT* and the *CASM22* RNAs (Figures 3C, 3D, and S5). Calculating the solvent-accessible surface area (SASA) of the two models, we found that the change in solvent accessible surface area ΔSASA (ΔSASA = SASA_CASM22_ -SASA_p53-WT_) between the CASM22 and the WT *p53* mRNA was −85.52 Å^2^. This implies that the CASM22 decreases the *p53* mRNA surface in contact with the solvent by forming more rigid 2D and 3D structures. The distance between the BOX-I stem-loop (red) and the first stem-loop (yellow) was significantly changed with the stem-loops being near to each other in the *p53*-*WT* mRNA with codon 22 (green) exposed. In the *CASM22* RNA, the stem-loops are split and codon 22 is buried. The 3D structures illustrate the conformational plasticity of the *p53* mRNA and how a single-nucleotide changes this feature.

### DNA damage induces *p53* mRNA structural changes

**Figure 4:**
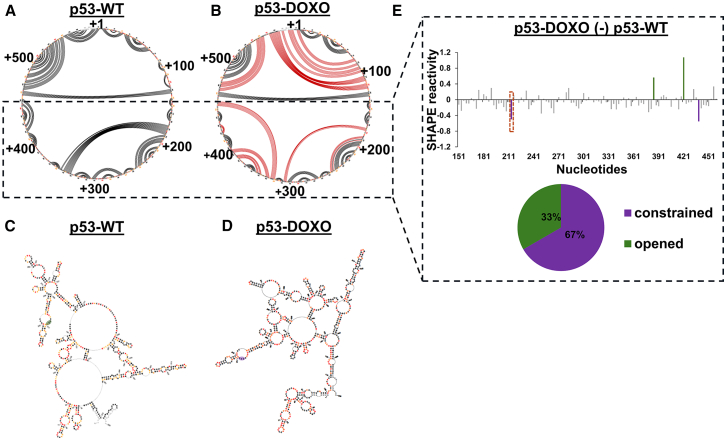
*p53* mRNA undergoes a structural switch in response to DNA damage Circular plot showing the base-pairing potentials of the *p53* mRNA based on the SHAPE reactivity under normal (A) and DNA damage conditions (B). Altered base-pairings under DNA damage conditions are indicated in red lines. Secondary structure of the *p53* mRNA coding sequence using the SuperFold algorithm based on the SHAPE values under normal (C) and DNA damage conditions (D). SHAPE-modified nucleotide sequences are indicated in orange/red. (E) Upper panel shows differential SHAPE reactivity of the *p53* mRNA under normal and DNA damage conditions. Windowed average reactivity calculated over 3 nucleotide sliding windows. (Lower panel) Pie chart shows the percentage of nucleotides constrained (violet) and opened (green) under DNA damage conditions. Constrained region at the 5′ part of the p53 CDS is indicated with red dashed box. See also Figure S4. For p53-WT, same representative secondary structure model was used both in Figures 3A and 4A. SHAPE-MaP data shown are representative of at least two independent repeats.

Having observed how the CASM22 prevents the formation of the downstream MDM2-binding platform by disrupting the *p53* mRNA dynamics, we next determined the *p53* mRNA structural changes following DNA damage. SHAPE-MaP was used to probe the *p53-WT* mRNA in response to DNA damage induced by doxorubicin (1 μM) treatment for 8 h. Interestingly, this did not result in significant changes in the BOX-I structure but resulted in major alterations in the base-pairing pattern both in the up- and downstream sequences of +120, indicated as red lines in the circular plots (Figures 4A–4D). The confidence levels of these base-pairing probabilities are illustrated in arc plots (Figure S4). While the overall difference in SHAPE reactivity was rather low between normal and DDR conditions, the region downstream of +120 (+151 to +460 nts) that participates in the formation of the MDM2-binding platform showed an overall decreased SHAPE activity with most of the nucleotides being constrained (67%) (Figure 4E).

### Localized perturbations in the BOX-I aptamer modulate downstream folding of p53 mRNA

**Figure 5:**
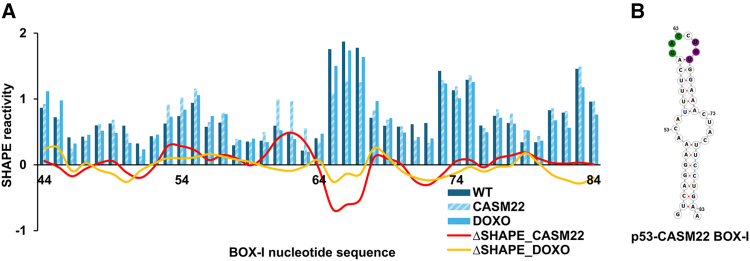
CASM22 targets BOX-I aptamer function (A) Comparison of BOX-I region SHAPE reactivity of the p53-*WT* mRNA under normal and DNA damage (DOXO) conditions and the *CASM22* mRNA. Windowed average reactivity, calculated over 3 nucleotide sliding windows shown. Red line indicates the differential SHAPE reactivity between CASM22 and WT; orange line indicates the differential SHAPE reactivity between normal and DNA damage conditions. (B) Secondary structure of BOX-I region of the *CASM22* mRNA, with superimposed ΔSHAPE (p53-CASM22 (-) p53-WT) sites. Green indicates highly reactive nucleotides in CASM22; violet indicates constrained nucleotides in CASM22. See also Figures S5 and S6. SHAPE-MaP data shown are representative of at least 2 independent repeats.

Despite no major alterations to the highly conserved BOX-I aptamer structure, SHAPE-MaP analysis revealed that CASM22 induces localized, yet significant, changes in SHAPE reactivity near codon 22. These perturbations disrupt interactions between BOX-I and adjacent regions, ultimately influencing the folding of downstream structural elements (Figure 5A). However, when we looked at BOX-I SHAPE reactivity, we observed that CASM22 causes a significant change at codon 22 (red curve) with a decrease in the reactivity around the CASM22 mutation site (+65 to +67) and an increase in reactivity at its flanking region (+61 to +63) (Figure 5B), consistent with CASM22 altering the interaction between the first stem-loop structure and the BOX-I aptamer (see also Figures 1 and 2).^13^ We did not observe any significant alterations in the SHAPE reactivity of BOX-I under the DNA damage conditions compared to normal conditions (orange curve, Figure 5A).

To address the putative role of cellular factors in the formation of the MDM2-binding platform during genotoxic stress, we carried out a hybrid *in vivo-in vitro* assay in which we isolated total RNA from cells treated, or not, with 1 μM doxorubicin for 8 h. After removal of proteins, we carried out 1M7 treatment and performed SHAPE-MaP. We observed that the RNA secondary structure of BOX-I aptamer from this hybrid assay looked similar to that of BOX-I *in vivo* with no significant alterations between normal and DNA damage conditions (Figures S6, S7A, and S7B). This indicates that the BOX-I aptamer does not interact with cellular factors that could interfere with the SHAPE reactivity and its structure is unchanged under different conditions. However, removal of proteins resulted in more flexible structures of downstream regions, both under normal and DDR conditions, showing that the stability of these structures depend on cellular factors (Figure S6). These observations support the notion that cellular factors do not interact with the BOX-I aptamer during genotoxic stress but are required to form a stable downstream MDM2-binding platform during RNA synthesis.

### MDM2 promotes the interaction between the *p53* mRNA and the ribosome

**Figure 6:**
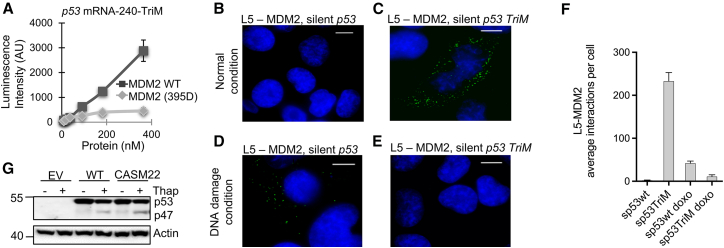
The *p53* mRNA-MDM2 interaction is sufficient to induce p53 synthesis (A) RNA ELISA with fixed amount (0.5 pmol) biotinylated 240 nts *p53* RNAs carrying synonymous mutations in codons 17, 18, and 19 (TriM) (see Figure S1) with increasing amount of recombinant wild-type MDM2 or MDM2(S395D). (B) Proximity ligation assays (PLA) (green dots) in cells treated with 10 μM MG132 using antibodies against MDM2 and ribosomal protein L5 in p53-null H1299 cells expressing a full-length p53 cDNA construct in which the AUG codons have been deleted (silent p53) to prevent interference from the p53 protein. (C) Same as in (B), but using a p53 cDNA expressing the silent *TriM* p53 mRNA. (D and E) Like in (B and C), but cells were treated with doxorubicin (DOXO) to activate the DNA damage response. (F) Summary of total number of PLA dots per cell corresponding to (B–E). (G) Western blot showing the expression of the p53 isoform p53/47 from wild-type or CASM22 *p53* mRNAs in H1299 cells treated with 10 μM MG132 under normal conditions or after activation of the UPR following thapsigargin (Thap) treatment. Actin was used as a loading control. The data represent at least three independent experiments. See also Figure S8. Scale bars represent 10 μm in (B–E).

Despite binding downstream of the second in-frame AUG (+118) of the *p53* mRNA, MDM2 promotes translation initiation from the +1 AUG. MDM2’s interactions with ribosomal factors and the *p53* mRNA are important for stimulating p53 synthesis.^28^ To test if the ATM kinase plays additional roles to promote p53 synthesis during the DDR, apart from promoting the MDM2-*p53* mRNA interaction, we used the *TriM p53* mRNA (SMs in codons 17, 18, and 19) (Figure S1). This RNA binds MDM2 WT, while the ATM phosphomimetic S395D mutation is required for MDM2 to bind the WT *p53* mRNA.^11^ Surprisingly, RNA ELISA assay showed that the *TriM p53* mRNA had poor affinity for the MDM2(S395D), as compared to MDM2 WT (Figure 6A). We took advantage of this, and we carried out *in cellulo* proximity ligation assays using antibodies against the large ribosomal subunit L5 and against MDM2 in H1299 p53 null cells expressing the *p53-WT* or *TriM* mRNAs. These RNAs lack the first, second, and third in-frame AUGs (silent *p53* RNAs) to prevent any effects related to the p53 protein. We observed that the WT *p53* mRNA promoted an interaction between MDM2 and the ribosome during DNA damage conditions (1 μM doxorubicin for 8 h) but not under normal conditions. The *TriM* mRNA, on the other hand, brought MDM2 to the ribosome under normal conditions, but not during DNA damage (Figures 6B–6F and S8). Together, these results show that the ATM-mediated induction of the MDM2-*p53* mRNA interaction is sufficient to bring MDM2 to the ribosome to stimulate p53 synthesis and does not require other ATM-dependent events.

Finally, we also wanted to know if the CASM22 specifically targets p53 DDR. The same region of the *p53* mRNA that promotes synthesis of full-length p53 during DDR also harbors the sequence that controls expression of the p53 isoform p53/p47 following PERK kinase activation during the UPR.^20^ However, treating cells with thapsigargin that induces the UPR did not affect the expression of p53/47 from the CASM22 RNA (Figure 6G). This demonstrates the specificity by which CASM22 targets the MDM2-binding structure of the *p53* mRNA during the DDR and highlights the dynamics of the *p53* mRNA in response to different signaling pathways.

## Discussion

**Figure 7:**
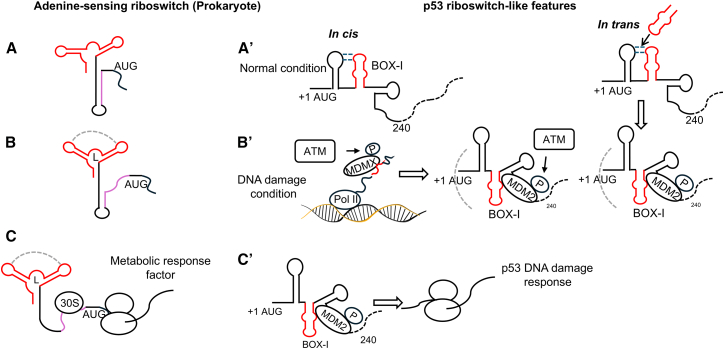
A model comparing similarities and differences between a prokaryotic riboswitch and the riboswitch-like features of the *p53* mRNA during the DNA damage response (in *cis*) or after addition of BOX-I oligo (in *trans*) (A) The adenine-sensing prokaryotic riboswitch is illustrated in “off” position.^31^^,^^32^ (A′) The *p53* mRNA 5′ coding sequence in “off” position under normal conditions with the first stem-loop interacting with the highly conserved second aptamer stem-loop (BOX-I, in red) preventing MDM2 binding.^13^ (B) Following ligand (L) binding, the riboswitch structure (in red) forms a compact kissing-loop structure. (B′) Following DNA damage, the ATM kinase phosphorylates MDMX and MDM2 at serines 403 and 395, respectively. The activated MDMX folds the nascent *p53* mRNA and releases BOX-I aptamer (red) from the first stem-loop to induce a compact downstream MDM2-binding platform. Similarly, adding free *BOX-I* RNA oligonucleotides to the *p53* mRNA in *trans* causes the BOX-I aptamer to dissociate from the first stem-loop. Both MDMX- and *BOX-I* oligonucleotide-mediated induction of the MDM2-binding platform are sensitive to the CASM22 and the DM nucleotide substitutions. (C) The riboswitch “on” structure exposes the Shine-Dalgarno sequence (purple) to the 30S pre-initiation complex to initiate synthesis of an enzyme to restore the metabolic status of the cell. (C′) MDM2 brings the *p53* mRNA to the ribosome to promote p53 synthesis and the consequent activation of p53 downstream DNA damage-controlling pathways.

This study illustrates riboswitch-like features within the *p53* mRNA that control p53 expression in response to DNA damage. The highly conserved BOX-I aptamer controls the folding of a downstream MDM2-binding platform, and this is disrupted by a single cancer-associated mutation in codon 22 (CASM22) of the BOX-I stem-loop. p53 is a key factor for maintaining the cellular homeostasis following DNA damage, and the fact that translation of the *p53* mRNA is directly responsive to the DNA damage-activated ATM kinase ensures that synthesis of the full-length p53 is synchronized with the genotoxic stress response. Unlike prokaryotic riboswitches that are generally located in the 5′ UTRs and are responsive to metabolites, the RNA sequences controlling p53 synthesis during genotoxic stress are within the coding sequence and controlled by a signaling pathway (Figure 7).^29^^,^^30^ Another difference is that the folding of the MDM2-binding platform takes place during synthesis by the ATM kinase-activated MDMX RNA chaperone. Hence, ATM activates both the *p53* mRNA chaperone MDMX and the translation stimulatory factor MDM2.

A further difference between prokaryotic riboswitches and the *p53* mRNA switch is that the latter involves longer sequences. This reflects the fact that the same RNA sequence takes on another conformation to control the initiation of the p53 isoform p53/47 from the second AUG at +118 by the PERK kinase during the UPR.^19^^,^^20^^,^^33^^,^^34^^,^^35^ Interestingly, the PERK response also involves sequences downstream of the +118 AUG, but the folding of this structure is not affected by the CASM22. This indicates that the CASM22 selectively disrupts the genotoxic stress response. The fact that sequences downstream of the +118 mediate both DDR and endoplasmic reticulum stress responses is reflected in the hybrid SHAPE-MaP data showing that RNA structures in regions downstream of +118 are dependent on cellular factors, while the conserved BOX-I structure is not. This is consistent with the notion that the BOX-I mediates its effect on the formation of the MDM2 platform during RNA synthesis. It should be noted that despite showing significant structural changes following DDR, the overall changes in free energy of the *p53* mRNA are relatively small, in line with the notion that the RNA can be folded into conformations with different structures and functions but with a similar free energy.

Prokaryotic riboswitches form tight 3D translation factor-interacting structures,^30^ and our RNA-RNA and RNA-protein interaction data show that the addition of the free *BOX-I* stem-loop RNA oligonucleotides (+45 to +83) results in the formation of a compact downstream MDM2-binding platform. Despite considerable efforts, we were unsuccessful in obtaining high-resolution cryo-EM structures of the +240 *p53* mRNA sequence, presumably due to the highly dynamic nature of the *p53* mRNA.^36^ However, the +1 to +120 nt sequence in the presence of 10 mM MgCl_2_ resulted in a low-resolution structure indicating the BOX-I stem-loop interacting with the first stem-loop (Figure S9). The interaction between these two stem-loops is supported by SHAPE-MaP reactivity analysis and solvent-accessible surface area predictions and by previous RNA footprint analysis predicting the interaction between codon 10 in stem-loop I and codon 21 in the BOX-I stem-loop (stem-loop II).^13^ This interaction is not evident in the SHAPE data, likely due to differences in the sensitivity and resolution of the two techniques, indicating the interaction is relatively weak or transient, in line with the notion that it is prevented by MDMX during the DDR. This suggests a model in which the formation of the MDM2-binding platform in the presence of free *BOX-I* RNA oligonucleotides involves dislodging the endogenous BOX-I stem-loop from the first stem-loop. This displacement would mimic the MDMX-mediated folding of the MDM2-binding platform during *p53* RNA synthesis.^13^ It is thus plausible that preventing the BOX-I from interacting with stem-loop I by MDMX is sufficient to form the MDM2-binding platform. The fact that the CASM22 and the DM have similar effects on MDMX-mediated RNA folding *in vitro* and free *BOX-I* -mediated folding *in vitro* strengthens the physiological relevance of the *BOX-I* as a DNA damage response RNA aptamer and indicates that MDMX and the free *BOX-I* induce the same MDM2-binding platform.

The BOX-I structure of the *p53* mRNA is highly conserved across species; the *p53* mRNA-MDM2 interaction also takes place in pre-vertebrates, and in the case of *Ciona intestinalies,* it is regulated by temperature.^37^ Hence, the *p53* mRNA switch has evolved from being controlled by physical conditions in pre-vertebrates to signaling pathways in mammalian cells.

By using the *TriM* message that binds MDM2 under normal conditions, but not during the DDR, we could show that ATM-mediated induction of the *p53* mRNA-MDM2 complex is sufficient to bring MDM2 to the ribosome and stimulates p53 synthesis.^28^^,^^38^ In addition to controlling translation initiation under different conditions, *p53* mRNA structures also play a role in linking p53 upstream-sensing pathways with downstream effector pathways. The ATM kinase interacts with the *p53* mRNA under normal conditions but is released from the *p53* mRNA during DNA damage.^39^ Furthermore, the *p53* mRNA also governs the phosphorylation of the nascent p53 protein at serine 15,^28^ which is a key event in p53’s activation toward the DDR response. Together, these different observations lead to the intriguing suggestion that the *p53* mRNA plays a key role in the different steps of p53 activation: from DNA damage sensing to the expression of a p53 protein committed to the DNA damage repair pathway.

The p53 protein provides a nuanced response repertoire to impose diverse and suitable cellular effects in response to specific stresses and damages. For this reason, the p53 protein carries intrinsically disordered regions, and its interactome with co-factors is governed by a large number of post-translational modifications.^40^^,^^41^ It is interesting that the diversity of the p53 response is further determined by signaling pathways governing the folding of intrinsically disordered mRNA sequences. It is likely that the role of the *p53* mRNA in managing the function of the encoded protein is not unique but instead reflects a broader concept by which mammalian riboswitch-like structures control gene expression and activity of the encoded protein in response to signaling pathways. It will be interesting to know if such structures are also targeted by SMs.

### Limitations of the study

Obtaining the 3D structure of the BOX-I + 240 nt ± MDM2 395D complexes would be highly desirable to enable direct visualization of the assembly. However, our attempts using cryo-EM were unsuccessful due to sample heterogeneity.

## Resource availability

### Lead contact

Requests for further information and resources should be directed to and will be fulfilled by the lead contact, Robin Fahraeus (robin.fahraeus@umu.se).

### Materials availability

This study did not generate any new unique reagents. Plasmids used in this study are available from the lead contact upon request.

### Data and code availability

• All data reported in this paper are available from the lead contact upon request; SHAPE-MaP raw data are available at https://github.com/medbioumu/CASM22-riboswitch.
• This paper does not report original codes.
• Any additional information required to reanalyze the data reported in this paper is available in Data S1 and from the lead contact upon request.

## Acknowledgments

This work was supported by Cancerforskningsfonden Norr (LP 24–2351; LP 24–2375), 10.13039/501100002794Cancerfonden (22 2505 Pj 01H), Vetenskapsradet (2022-01080), and the 10.13039/501100001824Czech Science Foundation, project no. 23-06884S and MH CZ—DRO (MMCI, 00209805). We would like to thank Michael Hall, Camilla Holmlund at the SciLifeLab National Cryo-EM facility, and Microscopy Infrastructure Umea for cryo-EM technical support and Tim Schulte and Piotr Drackowski for cryo-EM data processing support.

## Author contributions

RNA coIP and gel shift assays, S.C.; computational modeling and data analysis, L.W.; PLA and data analysis, K.K.; RNA ELISA and coIP, L.M.-C. and V.O.-I.; SHAPE-MaP experiment, data analysis, and manuscript writing, S.V.G.; supervision, study design, and manuscript writing, R.F. All the authors contributed to figure assembly and manuscript writing and review.

## Declaration of interests

The authors declare no competing interests.

## STAR★Methods

### Key resources table

**Table:** 

REAGENT or RESOURCE	SOURCE	IDENTIFIER
**Antibodies**		

Rabbit polyclonal anti-p53 (CM1)	Labome	Cat# 925401
HRP-linked anti-p53 (DO-1)	Abcam	Cat# ab204452
Mouse anti-MDM2 mAb (4B2)	Provided by Dr. Vojtesek	N/A
Mouse monoclonal anti actin (AC-15)	Thermo Fisher Scientific	Cat# AM4302
Goat anti-Rabbit HRP-conj.	Thermo Fisher Scientific	Cat# 32260
Goat anti-Mouse HRP-conj.	Thermo Fisher Scientific	Cat# G-21040
Rabbit anti-RPL5	Invitrogen	Cat# PA5-27539
Recombinant 6x His tagged protein	Clontech	Cat # 32106

**Bacterial and virus strains**		

BL21(DE)pLysS	Thermo Fisher Scientific	Cat# C606010

**Chemicals, peptides, and recombinant proteins**		

10% Mini-PROTEAN	Bio-Rad	Cat#: 4568034
1-Methyl-7-nitroisatoic anhydride	Sigma-Aldrich	Cat# 908401-250MG
Agencourt AMPure XP beads	Beckman Coulter	Cat# A63880
Amplicon Tagment Mix	Illumina	Cat# FC-131-1024
BSA	Sigmaaldrich	Cat# A3311
Chloroform	VWR	Cat# 0757
Complete protease inhibitor cocktail	Sigma-Aldrich	Cat#: 4693132001
DAPI	Thermo Fisher Scientific	Cat# D1306
Deoxynucleotide triphosphates (dNTPs)	New England Biolabs	Cat# N0447S
Dithiothreitol (DTT)	Fisher Bioreagents	Cat# BP172-5
DMSO	Sigma-Aldrich	Cat# D2438
Doxorubicin	Sigma-Aldrich	Cat# D1515-10mg
EDTA, pH 8.0	Thermo Fisher Scientific	Cat# AM9260G
FBS	Thermo Fisher Scientific	Cat# A3160502
Gelred Nucleic Acid Gel Stain	VWR	Cat# 41003
GeneJuice	Sigma-Aldrich	Cat# 70967-3
HEPES	Fisher Bioreagents	Cat# BP310-500
Immun-Blot Low Flurescence PVDF Paper Sets	Bio-Rad	Cat# 1620260
INTERFERin Kit	VWR	Cat# 101000036
L-glutamine	Thermo Fisher Scientific	Cat# 25030081
Magnesium chloride (MgCl2)	Thermo Fisher Scientific	Cat# AM9530G
Manganese chloride (MnCl2)	Fisher Bioreagents	Cat# BP541-100
MG-132	Selleck Chemicals	Cat# S2619
mMessage mMachine T7 Transcription Kit	Thermo Fisher Scientific	Cat# AM1344
Nextera XT Index Primers	Illumina	Cat# FC-131-1002
Nextera XT PCR Master Mix	Illumina	Cat# FC-131-1024
PageRuler Plus Protein Ladder	Thermo Fisher Scientifc	Cat# 26619
PBS	Merck	Cat# P2272
Phusion Flash High Fidelity PCR Master Mix	Thermo Scientific	Cat# F548L
Potassium chloride (KCl)	Thermo Fisher Scientific	Cat# AM9640G
Protein G Sepharose 4	Sigma-Aldrich	Cat# GE17-0618-01
Q5 Hot Start High-Fidelity DNA Polymerase	New England Biolabs	Cat# M0493S
QuikChange Lightning Muiti Site-Directed Mutagenesis Kit	Agilent Technologies	Cat# 210515
RiboLoc RNAase Inhibitor	Thermo Fisher Scientific	Cat # EO0381
RIPA buffer	Thermo Fisher Scientific	Cat# 89900
RNA clean and concentrator kit	Nordic BIOSITE	Cat# R1013
RNase-out	Invitrogen	Cat# 10777019
RPMI 1640 medium	Thermo Fisher Scientific	Cat# 31870074
Saponin	Merck	Cat# 558255
Sodium chloride (NaCl)	Thermo Fisher Scientific	Cat# AM9760G
Streptavidin	New England Biolabs	Cat # N7021S
Streptomycin	Thermo Fisher Scientific	Cat# 15140122
SuperScript II reverse transcriptase	Thermo Fisher Scientific	Cat# 18064-014
SYBR Green Master	Thermo Fisher Scientific	Cat# A257
Tagment DNA Buffer	Illumina	Cat# FC-131-1024
Thapsigargin	Thermo Fisher Scientific	Cat# T7459
Tris, pH 8.0	Thermo Fisher Scientific	Cat# AM9850G
Trizol Reagent	Thermo Fisher Scientific	Cat# 15596026
Trypsin-EDTA (0.25%)	Thermo Scientific	Cat# 25200072
Turbo DNase (2 U/μl)	Thermo Fisher Scientific	Cat# AM2238
Turbo DNase Reaction Buffer (10×)	Thermo Fisher Scientific	Cat# AM2238
Yeast tRNA	Invitrogen	Cat# 10618255
96-well Microplate Nunc maxisorp White	Thermo Fisher Scientific, Nunc	Cat # 436110

**Critical commercial assays**		

RNA 3' End Biotinylation Kit	Pierce Antibodies	Cat # 20160
Qubit dsDNA High Sensitivity assay kit	Thermo Fisher Scientific	Cat# Q32854
Bioanalyzer High Sensitivity DNA kit	Agilent Technologies	Cat# 5067-4626
PureLink PCR Micro kit	Thermo Fisher Scientific	Cat# K310250

**Deposited data**		

ShapeMapper reactivity profiles	https://github.com/medbioumu/CASM22-riboswitch	

**Experimental models: Cell lines**		

H1299 (human non-small cells lung cancer, not expressing p53	ATCC	Cat# CRL-5803

**Oligonucleotides**		

BOX I WT: AGUCAGGAAACAUUUUCA GACCUAUGGAAACUACUUCCU	Eurofins	N/A
Primers for pcDNA3-p53-CASM22/TriM-240: p53 CASM: CATTTTCAGACCTGTGGAAACTACTTCC p53 TriM: CTCTGAGTCAGGAGACCTTCTCAGACCTATGGA	Eurofins	N/A
Primers for Silent p53TriM (pcDNA3-sp53TriM): 1. p53TriM44A-F GCAATGGCTGCTGCG GCGCTGTCCCCGGACGAT 2. p53TriM44A-R ATCGTCCGGGGACAG CGCCGCAGCAGCCATTGC 3. p53TriM44D-F GCAATGGCTGCTGCG GATCTGTCCCCGGACGAT 4. p53TriM44D-R ATCGTCCGGGGACA GATCCGCAGCAGCCATTGC	Eurofins	N/A
Mdm2S394D-F CCCAGGAGAGTGACGACTATGACCA ACCAATCGACTTCCAGC Mdm2S394D-R GCTGGAAGTCGATGGTTGGTCATA GTCGTCACTCTCCTGGG	Eurofins	N/A
Oligonucleotides used for SHAPE-MaP library preparation. 1.P53-RT primer TCCACTCGGATAAGATGCT 2. p53_forward ATGGAGGAGCCGCAGTCAGAT 3. p53_reverse TCCACTCGGATAAGATGCT	Eurofins	N/A

**Recombinant DNA**		

pcDNA3-p53-WT-FL	Provided by Dr. Malbert-Colas	N/A
pcDNA3-p53-CASM22-FL	Provided by Dr. Malbert-Colas	N/A
pcDNA3-p53-DM-FL	Provided by Dr. Malbert-Colas	N/A
Silent p53WT (pcDNA3-sp53WT)	Provided by Dr. Malbert-Colas	N/A
Silent p53TriM (pcDNA3-sp53TriM)	Provided by Dr. Malbert-Colas	N/A
pcDNA3-MDM2 S395D-FL	Provided by Dr. Malbert-Colas	N/A
pcDNA3-p53-WT-120	Provided by Dr. Malbert-Colas	N/A
pcDNA3-p53-WT-240	In house	N/A
pcDNA3-p53-CASM22-240	In house	N/A
pcDNA3-p53-DM-240	In house	N/A
pcDNA3-p53-TriM-240	In house	N/A
Pet-28a (+) -MDM2 395D	Provided by Dr. Malbert-Colas	N/A

**Software and algorithms**		

Image lab	Bio-Lab	https://www.bio-rad.com/en-se/product/image-lab-software
Prism 5	GraphPad	https://www.graphpad.com
ShapeMapper 2.1.3	https://webshare.oasis.unc.edu/weeksgroup/shapemapper-2.1.3.tar.gz	
Superfold_v1.0	https://webshare.oasis.unc.edu/weeksgroup/Superfold_v1.0.tar.gz	
PyMOL	https://github.com/schrodinger/pymol-open-source	
RNAComposer	https://rnacomposer.cs.put.poznan.pl/	

### Experimental model and study participant details

#### Cell culture

p53-null H1299 cells (human non-small-cell lung carcinoma) were cultured in RPMI 1640 medium supplemented with 10% fetal bovine serum, 100 U/mL penicillin, 100 μg/mL streptomycin, and 2 mM L-glutamine, and maintained at 37°C in a humidified 5% CO2 incubator. Plasmid DNA and 39-mer oligonucleotide transfections were carried out in a 6-well culture plate. For each plasmid DNA transfection, 3 μL of GeneJuice reagent was mixed with 100 μL of serum-free medium. After 5 minutes of incubation at room temperature, 100 ng of plasmid DNA was added to the mixture and incubated for 15 minutes. The final mixture was added dropwise to the cells. The second co-transfection with Locked Nucleic Acid (LNA) 39-mer oligonucleotides was carried out 12 hours after plasmid DNA transfection using the INTERFERin kit. For each well, 3 pmol of 39-mer oligonucleotide RNA was diluted with 100 μL medium without serum. 2 μL of INTERFERin reagent was added to the mixture and incubated for 10 minutes before adding it to the cells. DNA damage was induced by treating cells with the indicated concentration of doxorubicin prepared in DMSO for 8 hours, unless specified otherwise. MG-132 was added to the cells two hours before harvesting.

### Method details

#### Western blotting

Cells were lysed in RIPA buffer (Thermo Fisher Scientific) with a complete protease inhibitor cocktail (Sigma-Aldrich). For Western blot analysis, 10 μg of protein was loaded onto a 10% Stain-Free gel (Bio-Rad) and transferred to a Low Fluorescence PVDF membrane (Bio-Rad). Total protein detection was performed using Image Lab software on the ChemiDoc Touch Imaging System (Bio-Rad). p53 protein was detected by probing with HRP-linked anti-p53 DO-1 antibody.

#### In vitro transcription

*In vitro* transcription was carried out according to the manufacturer’s instructions for the mMESSAGE mMACHINE T7 Transcription Kit with some modifications. Briefly, 1 μg of linearized wild-type or mutant p53 (CASM22, TriM, or DM) DNA constructs were used as templates, and mRNA synthesis was carried out at 37°C overnight. RNAs were purified using the RNA Clean & Concentrate Kit (ZYMO Research) and quantified by NanoDrop.

#### RNA gel shift

The p53 mRNAs were heated at 90°C for 3 minutes, followed by cooling on ice for 5 minutes. They were refolded with RNA folding buffer (20 mM Tris-HCl, pH 8.0, 0.01 mM ZnSO_4_, 150 mM NaCl, 2 mM MgCl_2_) at room temperature for 10 minutes. The *BOX-I* RNA oligonucleotides, heated at 90°C for 1 minute and then cooled on ice for 5 minutes, were added and incubated for an additional 15 minutes. The RNA mixture was loaded onto a 1% agarose gel and electrophoresed on ice. Bands were visualized by post-staining using GelRed at room temperature.

#### In vitro quantitative RNA Co-Immunoprecipitation (Co-IP)

*In vitro* RNA Co-IP was carried out as previously described with slight modifications. In brief, +240 nts p53 or +120 nts *p53* RNAs were co-incubated with MDM2(S395D) protein at a 1:40 ratio in binding buffer (50 mM Tris, pH 7.5, 150 mM NaCl, 0.01 mM ZnSO_4_, 2 mM MgCl_2_, 0.02 mg/mL yeast tRNA, 0.2 mg/mL BSA) at room temperature for 30 minutes. BOX-I RNA oligonucleotides were introduced to the *p53* RNA and MDM2 protein mixture, followed by incubation at room temperature for another 15 minutes. MDM2–RNA complexes were pulled down at +4°C overnight using 4B2 anti-MDM2 mAb, and bound RNAs were purified using TRIzol. The precipitated RNAs were analyzed by RT-qPCR, and the relative RNA quantification was presented as the fold change between complexes with and without BOX-I RNA oligonucleotides, plotted in a graph.

#### DNA constructs

pcDNA3-p53-CASM22-240, pcDNA3-p53-TriM-240 were generated from pcDNA3-p53-WT-240 by using the QuikChange Lightning Multi Site-Directed Mutagenesis Kit.

#### Protein expression and purification

BL21(DE3)pLys cells containing the pET-28(a)-MDM2 S395D plasmid DNA were grown in LBkm+ medium at 37°C until the O.D. = 0.6-0.8. Protein expression was induced by adding IPTG, and the culture was incubated at 16°C for 16 hours. Cells were harvested by centrifugation, and the pellet was lysed in lysis buffer (200 mM NaCl, 40 mM Imidazole, 10 μM ZnSO_4_, 50 mM Tris, pH 8.0, 10% Glycerol) with a protease inhibitor cocktail. Lysis was performed using sonication, and the insoluble fraction was resuspended in 8M urea buffer (8M Urea, 150 mM NaCl, 10 mM Imidazole, 50 mM Tris, pH 8.0). Protein purification was carried out using a Ni-NTA affinity column. The bound protein was washed and eluted in elution buffer (200 mM NaCl, 300 mM Imidazole, 10 μM ZnSO_4_, 50 mM Tris, pH 8.0). Finally, the eluted protein was dialyzed overnight at 4°C in dialysis buffer (150 mM NaCl, 10 μM ZnSO_4_, 20 mM Tris, pH 8.0).

#### Protein-RNA ELISA

96-well plates were coated with streptavidin (100 μg/mL) in 0.1 M NaHCO_3_ (50 μL/well) overnight at 4°C. After incubation, plates were washed 6 times with 200 μL of 0.1% PBS-Tween and blocked with 1000 μL of 3% BSA, 0.1 μg/mL of streptavidin in PBS overnight at 4°C. The plates were washed 6 times with 0.1% PBS-Tween. A mix of biotinylated *p53* mRNA (0.5 pmol) (corresponding to nts +1 to +240) and indicated proteins was incubated in binding buffer (50 mM Tris, pH 7.5, 150 mM NaCl, 0.02 mg/mL yeast tRNA, 0.2 mg/mL BSA) for 30 minutes at 37°C, then added to the plates (50 μL/well) and incubated for 1 hour at RT. The plates were washed 6 times with 200 μL/well of 0.1% PBS-Tween, then 50 μL of anti-MDM2 4B2 mAb (PBS 1:1000) was added (50 μL/well) and incubated for 1 hour at RT. Plates were washed 6 times with 200 μL/well of 0.1% PBS-Tween, then 50 μL of ECL mix was added, and luminescence was measured.

#### SHAPE-MaP

The RNA SHAPE-MaP was performed as described previously.^20^^,^^24^ Briefly, H1299 cells grown in 6-well plates were transiently transfected with the indicated constructs. 36 hours post-transfection, cells were washed with PBS and 900 μL of RPMI media was added. The SHAPE reagent 1-Methyl-7-nitroisatoic anhydride (1M7) (Sigma-Aldrich) was dissolved in DMSO and added to a final concentration of 10 mM in RPMI media for ∼90 seconds at 37°C. The same volume of DMSO was added to the unmodified samples. Cells were washed with PBS and harvested. RNA purification was carried out using the RNeasy kit (Qiagen), followed by DNase I digestion for 30 minutes at 37°C. Reverse transcription of purified RNA was carried out with the primers indicated in the Data S1, using MaP buffer and Superscript II Reverse Transcriptase. Synthesized cDNAs were purified and amplified using Q5 DNA polymerase (NEB) with the indicated p53 primers. PCR products were purified and quantified with a Qubit fluorometer and diluted to 0.2 ng/μL. Purified amplicons were tagmented, and the library was created using the Illumina Nextera PCR library kit. Products from library PCR were purified with the Agencourt AMPure XP beads as described.^24^ Library concentration was measured with a Qubit fluorometer, and the size distribution was measured using an Agilent 2100 Bioanalyzer according to the manufacturer’s instructions. Libraries were sequenced with the NovaSeq System, Paired-end 150 (NovaSeq PE150, Novogene, UK). SHAPE reactivity profiles and comparisons were generated using the ShapeMapper 2 and deltaSHAPE scripts, with default settings, and aligned to the indicated p53 sequences. SHAPE-Map data shown are representative of at least two independent biological repeats. SHAPE reactivity variations across biological repeats of each experiment were examined using Spearman’s rank-order correlation coefficient (see Data S1).

#### RNA structure analysis and computational models

Superfold was used to integrate SHAPE-MaP data to model RNA secondary structures. Based on the predicted secondary structure, RNAeval from the ViennaRNA Package^25^ was used to evaluate the free energy of the *p53* mRNA, and RNAComposer^26^^,^^27^ was applied to predict a 3D structure of the *p53* mRNA. PyMOL 2.4 was used to visualize the conformations and calculate the solvent-accessible surface area (SASA) of the *p53* mRNA structure.

#### Proximity ligation assay (PLA)

H1299 (human non-small cell lung cancer, p53 null) cells were incubated at 37°C, 5% CO_2_ in RPMI medium supplemented with antibiotics, 2 mM L-glutamine (Gibco/Invitrogen), and 10% fetal serum (Hyclone) on sterilized transparent 24-well plates. They were transfected with either silent p53WT (pcDNA3-sp53WT) or silent p53TriM (pcDNA3-sp53TriM) constructs. Treatment was done with either DMSO (negative control) or 1 μM Doxorubicin (Sigma) for 2 hours, and cells were fixed in 4% PFA. After three washes with PBS for 10 minutes, the samples were incubated with blocking buffer (3% BSA, 0.1% saponin in PBS). The samples were then incubated with primary antibodies for 2 hours at room temperature. The antibodies used were: the 4B2 anti-MDM2 mouse (a kind gift from Dr. B. Vojtesek) and the rabbit anti-RPL5 (PA5-27539; Invitrogen). All antibodies were used at a dilution of 1:200. The PLA Duolink RED kit (Sigma-Aldrich) was used. Images were captured using 640 nm filters on a standard microscope. DAPI staining determines nuclear signals. For each sample, dots were counted in 50 randomly selected cells during the imaging. This procedure was repeated in at least three independent experiments. The values were used to prepare a graph using GraphPad Prism 5 software. Scale bars represent 10 μm.

### Quantification and statistical analysis

Relative quantification of RNA-Co-IP qRT-PCR (Figure 1B) was calculated as fold changes between complexes with and without *BOX-I* RNA Oligonucleotide; RNA quantification in ELISA gel shifts (Figures 1C, 1E–1H, and S2) and Western Blot quantification (Figure 1J) were performed using Image Lab software. All experiments were repeated at least three times, and statistical analysis was performed using Student's t-test.

## Supplemental information

Document S2. Supplementary information methods

Data S1. Supporting data sets
