# Supplementary material for: The p53 mRNA exhibits riboswitch-like features under DNA damage conditions
Source: iScience. 2025 Sep 12;28(10):113555. doi: 10.1016/j.isci.2025.113555 (PMC12510224; doi:10.1016/j.isci.2025.113555)
Supplement: Document S1. Figures S1–S9 [file mmc1.pdf]

iScience, Volume *28*

Supplemental information

The *p53* mRNA exhibits riboswitch-like features under DNA damage conditions

Sa Chen, Lixiao Wang, Laurence Malbert-Colas, Konstantinos Karakostis, Vanesa Olivares-Illana, Sivakumar Vadivel Gnanasundram, and Robin Fahraeus

p53 WT
+1
UGGAGGAGCC**G**CAGUCAGAUCCUAGCGUCGAGCCCCCUCUGAG**UCAGGAAACAUUUUCAGACCUAUGGAAACUACUUCCUGA**AAACAACGUUCUGUCCCCCUUGCCGUCCCAAGCAAUGGAU
GAUUUGAUGCUGUCCCCGGACGAUAUUGAACAAUGGUUCACUGAAGACCCAGGUCCAGAUGAAGCUCCCAGAAUGCCAGAGGCUGCUCCCCCCGUGGCCCCUGCACCAGCAGCUCCU +240

p53 CASM22
AUGGAGGAGCCGCAGUCAGAUCCUAGCGUCGAGCCCCCUCUGAG**UCAGGAAACAUUUUCAGACCUGUGGAAACUACUUCCUGA**AAACAACGUUCUGUCCCCCUUGCCGUCCCAAGCAAUGGA
UGAUUUGAUGCUGUCCCCGGACGAUAUUGAACAAUGGUUCACUGAAGACCCAGGUCCAGAUGAAGCUCCCAGAAUGCCAGAGGCUGCUCCCCCCGUGGCCCCUGCACCAGCAGCUCCU

p53 DM
AUGGAGGAGCCGCAGUCAGAUCCUAGCGUCGAGCCCCCUCUGAG**UCAGGAAACAUUUUCAGACCUGUGGAAACUACUUCCUGA**AAACAACGUUCUGUCCCCCUUGCCGUCCCAAGCAAUGGA
CGAUUUGAUGCUGUCCCCGGACGAUAUUGAACAAUGGUUCACUGAAGACCCAGGUCCAGAUGAAGCUCCCAGAAUGCCAGAGGCUGCUCCCCCCGUGGCCCCUGCACCAGCAGCUCCU

p53 TriM
AUGGAGGAGCCGCAGUCAGAUCCUAGCGUCGAGCCCCCUCUGAG**UCAGGAGACCUUCUCAGACCUAUGGAAACUACUUCCUGA**AAACAACGUUCUGUCCCCCUUGCCGUCCCAAGCAAUGGA
UGAUUUGAUGCUGUCCCCGGACGAUAUUGAACAAUGGUUCACUGAAGACCCAGGUCCAGAUGAAGCUCCCAGAAUGCCAGAGGCUGCUCCCCCCGUGGCCCCUGCACCAGCAGCUCCU

Fig. S1. The 240 nts. *p53* mRNA sequences: The *BOX-I* sequence is highlighted in bold and the mutatant nucleotides are shown in red.

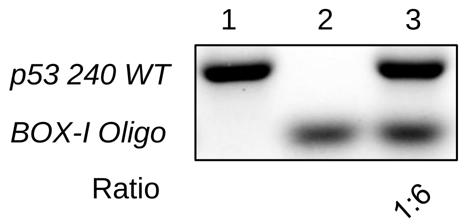

Fig. S2. Analysis of *p53 240* RNA and *BOX-I* Oligo interactions by denature agarose gel electrophoresis. Lane 1: *p53 240* RNA alone; Lane 2: *BOX-I* Oligo alone; Lane 3: Mixture of *p53 240* RNA and *BOX-I* Oligo in the ratio 1:6. RNAs were mixed with formamide to a final concentration of 60% (w/v), then denatured by heating at 90°C for 3 minutes, followed by immediate chilling on ice for 3 minutes. Before loading, half of the sample volume of 50% Glycerol was added. Sample were run on an agarose gel in a ice bath at 90 V for 50 minutes. Gel was post-stained with GelRed for visualization. Related to main figure 1.

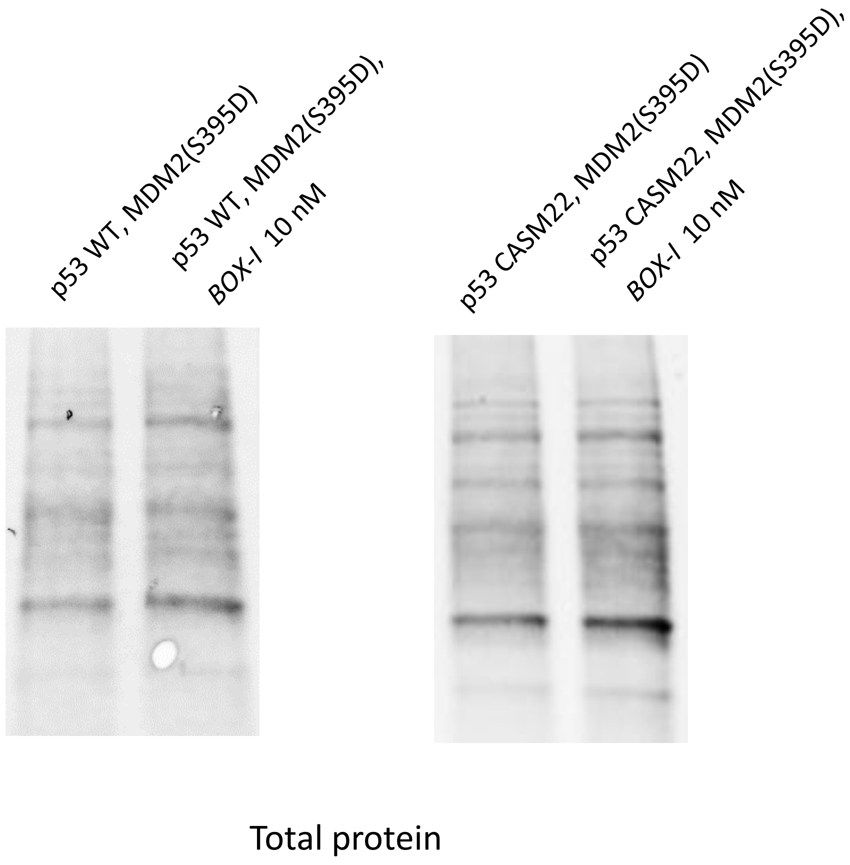

Fig. S3. The stain free blots (LF PVDF) visualize total protein and served as loading control for figure 2A. Values were normalized according to loading control of each experiment. Relates to main figure 2.

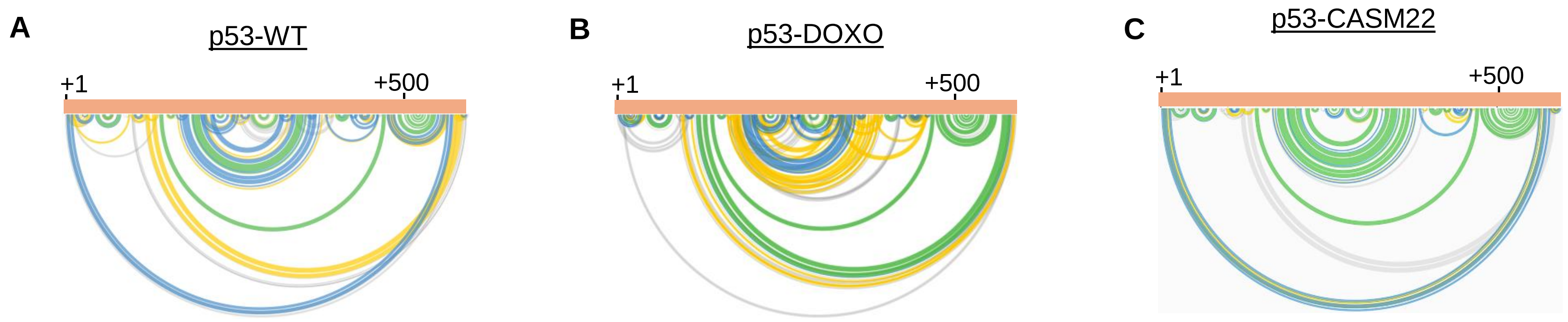

Fig. S4. Arc plots showing the significance and accuracy of the base pairing potentials observed in the circular plots for **A)** p53-WT, **B)** p53-DOXO, and **C)** p53-CASM22. Highly probable base-pairings are indicated in green color. Relates to main figures 2 and 3.

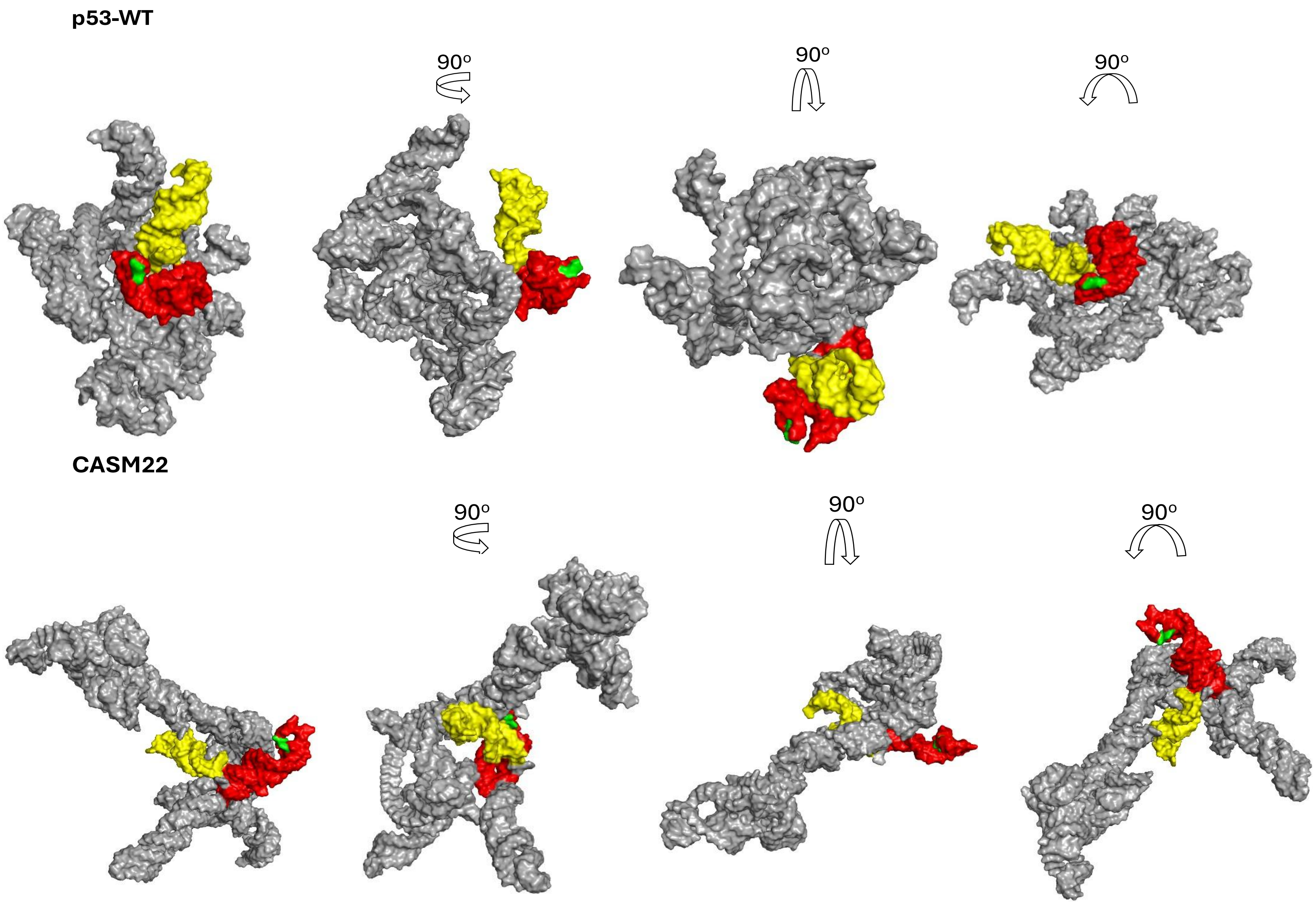

Fig. S5. Different views of the predicted 3D structures of P53-WT and CASM22, highlighting the first stem-loop (yellow), BOX-I (red), and the mutation position at L22L (green). Relates to main figures 2 and 4.

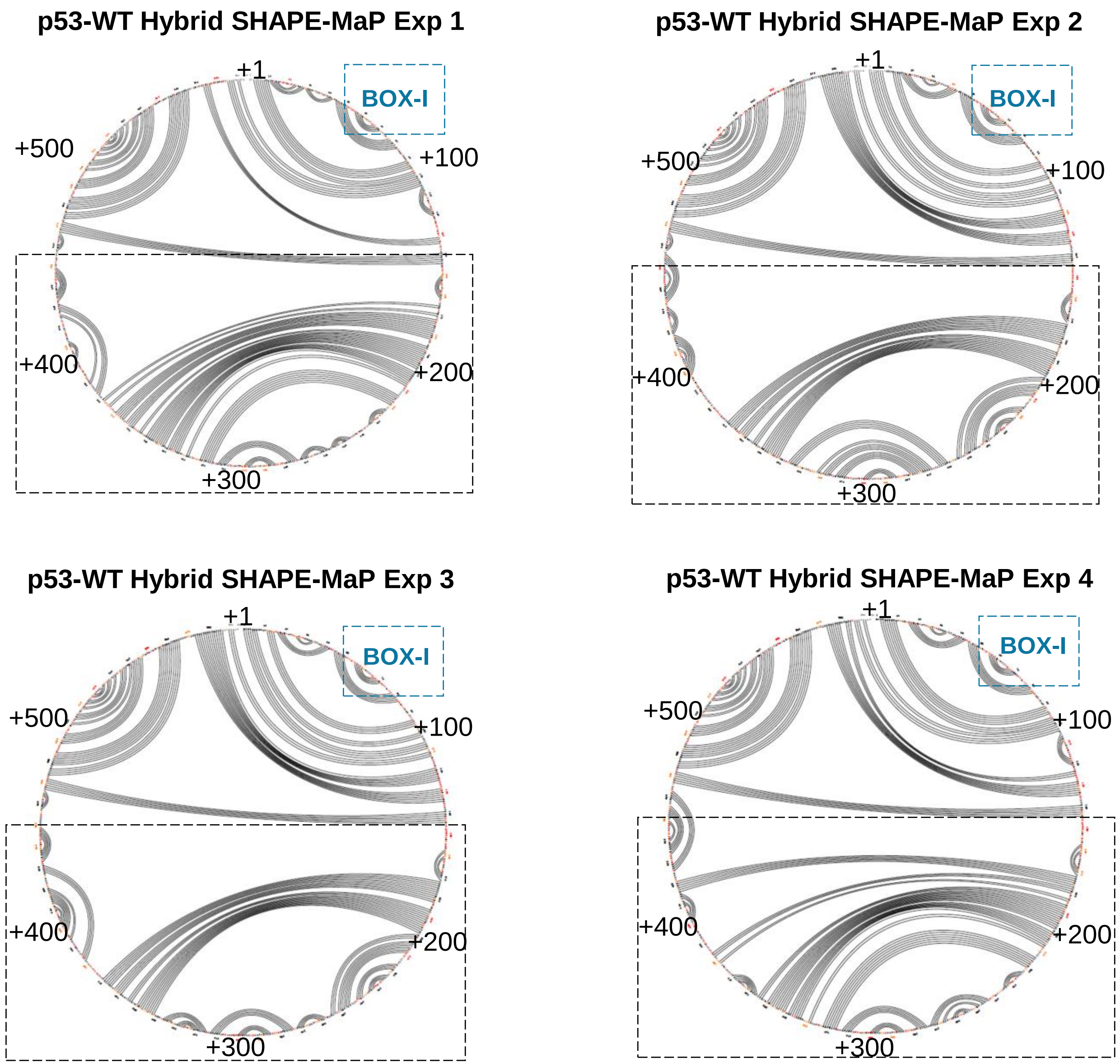

Fig. S6. Circos plots from independent experiments of *p53-WT* mRNA hybrid-SHAPE-MaP, showing the base pairing potentials of the *p53-WT* mRNA based on the SHAPE reactivity. RNA regions involved in DNA damage-mediated MDM2 binding platform (marked with black dashed box) exhibited structural variations across independent experiments of hybrid-SHAPE. Related to main figure 4.

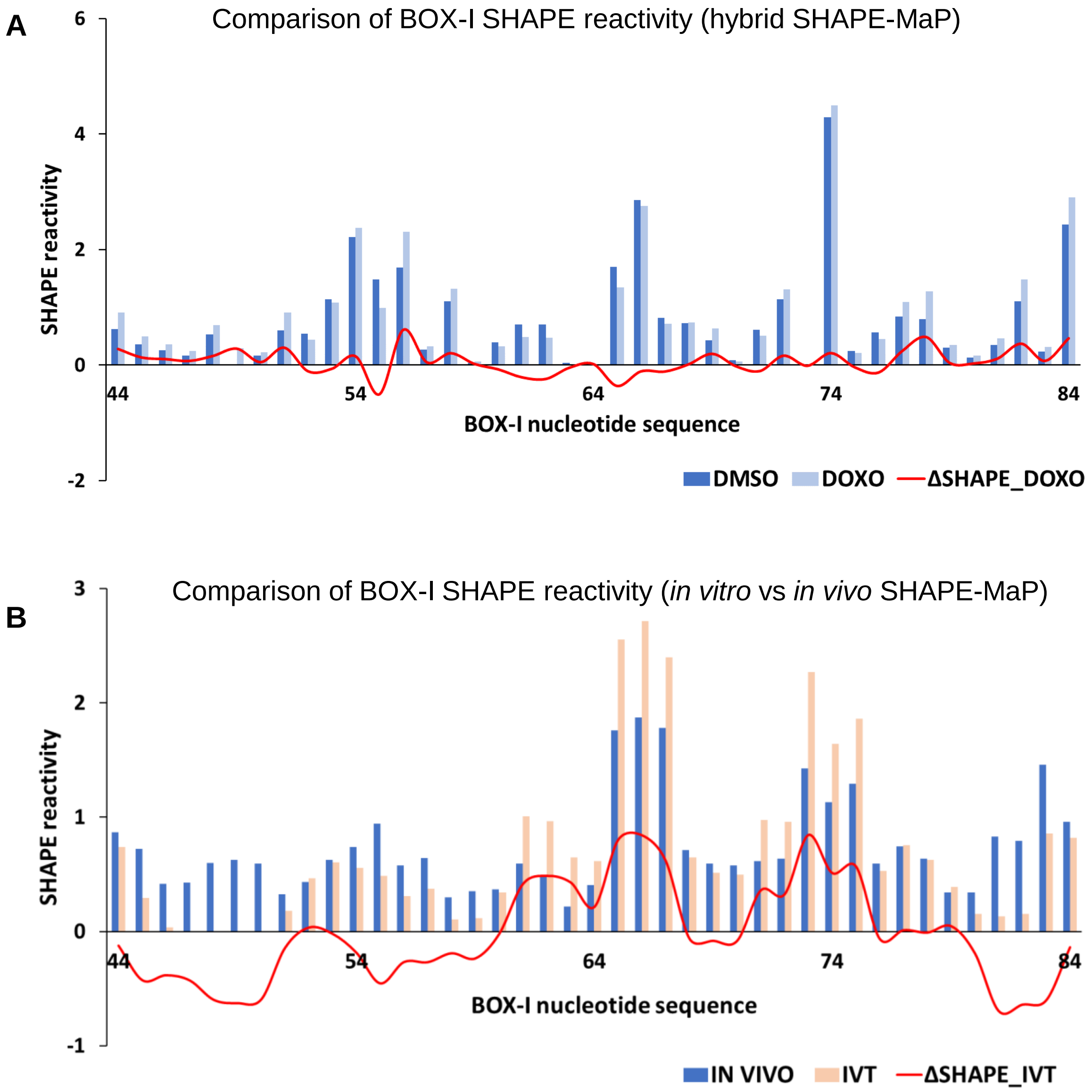

Fig. S7. A) Differential SHAPE reactivity of BOX-I aptamer under normal and DNA damage conditions of hybrid SHAPE-MaP, **B)** Differential SHAPE reactivity of BOX-I aptamer between *in vivo* and *in vitro* SHAPE-MaP of *p53-WT* mRNA. Windowed average reactivities calculated over 3-nt sliding windows. Related to main figures 2 and 4.

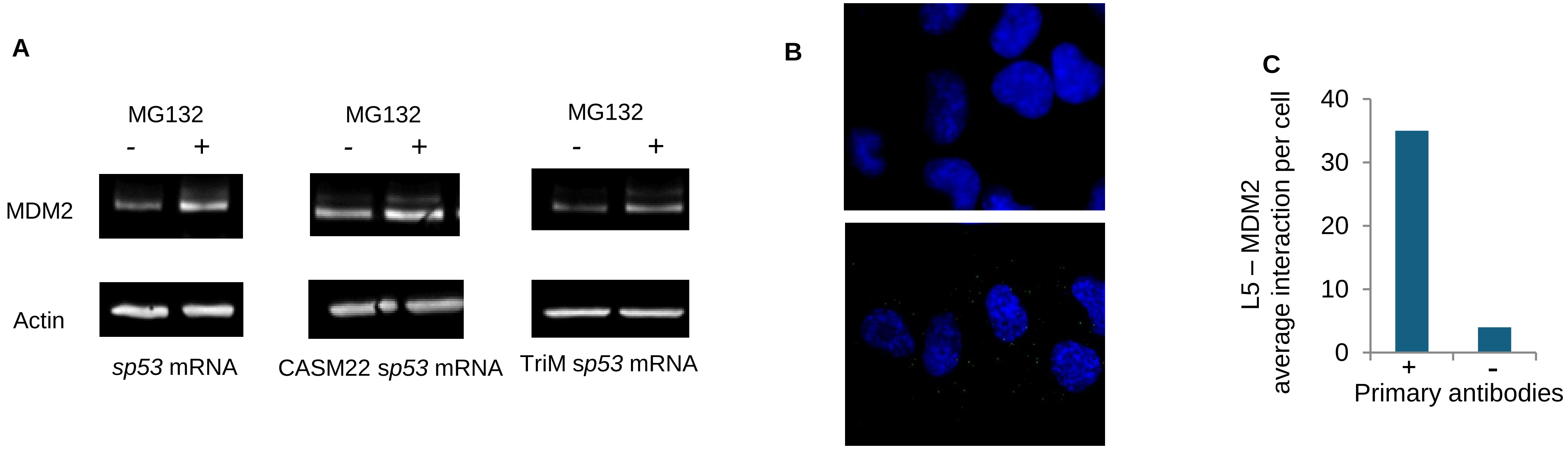

Fig. S8. A) The effect of the *p53* mRNA on MDM2 expression levels. Cells expressing indicated p53 mRNAs were treated with the proteasome inhibitor MG132 (10 μM), or not. **B)** PLA without (above) or with (below) primary antibodies against L5 – MDM2 in cells expressing Trim *sp53* RNA. **C**) The average L5 – MDM2 interactions in > 200 cells with and without primary antibodies. Relates to main figure 5.

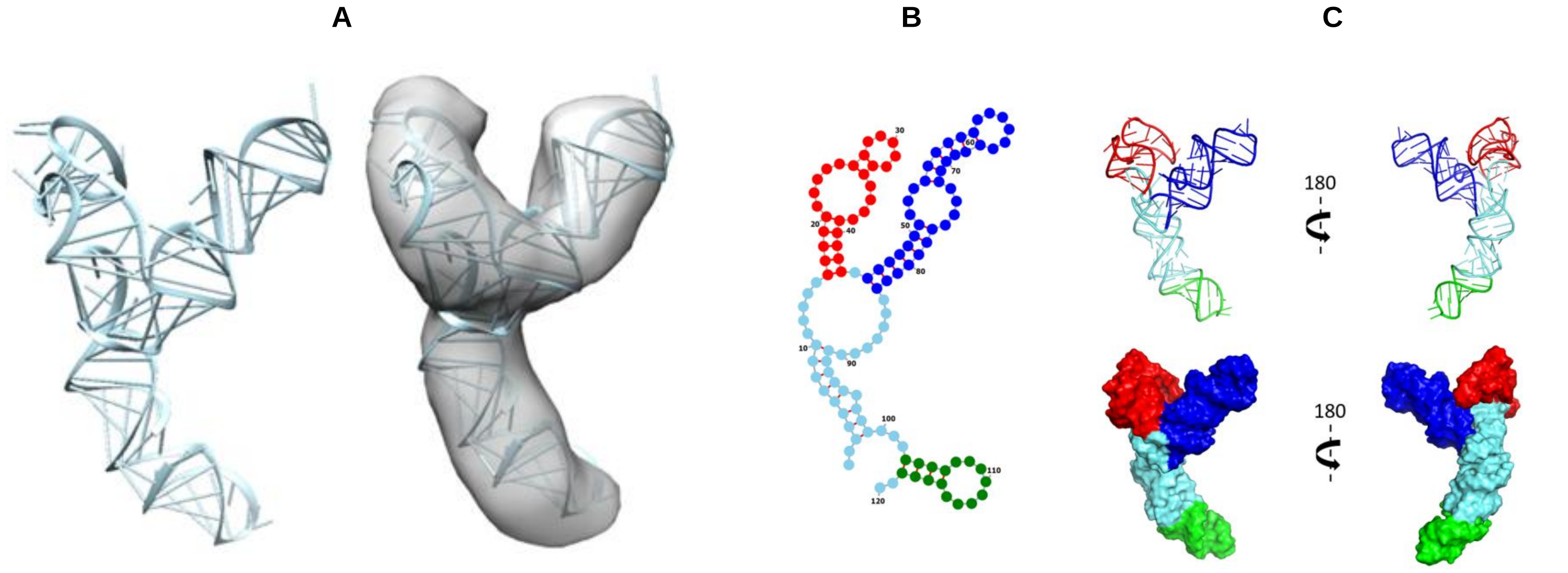

Fig. S9. A Cryo-EM density map. A) The secondary and tertiary structures of the *p53* mRNA. **B)** The secondary structure coloured by the different stem-loop, red:1st stem-loop, blue: BOX-I and green:3rd stem-loop. **C)** The 3D structure and surface view colored by the same scheme as in (**B**). Related to main figure 2.
