# Supplementary material for: The p53 mRNA exhibits riboswitch-like features under DNA damage conditions
Source: iScience. 2025 Sep 12;28(10):113555. doi: 10.1016/j.isci.2025.113555 (PMC12510224; doi:10.1016/j.isci.2025.113555)
Supplement: Document S2. Supplementary information methods [file mmc2.pdf]

Supplementary information_methods

Plasmid constructs and Oligonucleotides

p53-WT and p53-CASM22 constructs used for cell transfection were generated using the pcDNA3 eukaryotic expression vector (Life Technologies, Carlsbad,CA,USA) and have been described previously [1]. p53 240 and p53 120 DNA sequences were amplified from p53 WT-pcDNA3 construct and generated in pET-28a vector for *in vitro* transcription.

BOX-I RNA Oligonucleotide (LNA modified) used for cell transfection was purchased from QIAGEN (Cat. 339412 YCO0293244).

RNA preparation for CryO-EM

RNA (1-2 µg/µl) were heated at 90°C for 3 minutes, kept on ice for 5 minutes, then added RNA folding buffer (20 mM Tris-HCl, 10 µM ZnSO4, 150 mM NaCl, 2 mM $MgCl_2$), incubated at room temperature for 10 minutes. Plunge freeze immediately to grids.

Cryo-EM data acquisition

Cryo-EM data was collected using a Titan Krios microscope equipped with a Falcon4i direct electron detector at the Umeå centre for electron microscopy (UCEM) Cryo-EM Facility. Sample grids were imaged at 300 kV, with an intended defocus range of −1.5 to −3 µm with a magnification of X 165,000 in electron counting mode (0.704 Å per pixel), and at a dose rate of 15.77 e^-/Å^2/second. Movies were collected with a total dose of 45 electrons per Å^2. Movies were recorded by EPU v3.2.0 software. A total of 10,000 video stacks for +120 nts. of the p53 mRNA coding sequence were collected.

Single-particle image processing and 3D reconstruction

All data were processed by cryoSPARC 4.0[2]. After motion correction and CTF estimation, 8548 high quality movies were selected for further analysis. An initial stack of 658,630 particles were “blob picked” and extracted using an extraction box size 256 pixel and subjected to 2D classification. Through several rounds template-based particle picking, a total 96,958 particles were extracted and were then used to produce 3 ab initio models. Two rounds of heterogeneous refinement were performed to remove contaminant particles. The final subset of 39,008 particle images were selected for non-uniform refinement. RNA

conformational dynamics analysis was performed by the 3D Variability Analysis (3DVA)[3] module within the cryoSPARC program.

p53 mRNA model building

The p53 mRNA models were built with Rosetta autoDRRAFTER RNA modelling application[4 5]. AutoDRRAFTER generated 2000 models per round based on p53 mRNA sequences, its secondary structure by predicted RNAstructure[6] and cryo-EM density map as 3D experimental restraints. A total of six rounds of modelling were performed for the first +120 nts of the p53 mRNA coding sequence until the convergence value of the modle is less than 10 Å. The further refinement and model quality assessment were performed by using Phenix V1.2[7]. The final models were evaluated by MolProbity[8]. Molecular figures were prepared using Chimera[9] and PyMol[10]. Secondary structure diagrams were prepared with RNAStructure. All jobs were run on the cluster of the High-Performance Computing Center North (HPC2N) Sweden using the latest distribution of Rosetta and auto-DRRAFTER.

References.

1. Candeias, M.M. et al. P53 mRNA controls p53 activity by managing Mdm2 functions. *Nat Cell Biol* **10**, 1098-105 (2008).
2. Punjani, A., Rubinstein, J.L., Fleet, D.J. & Brubaker, M.A. cryoSPARC: algorithms for rapid unsupervised cryo-EM structure determination. *Nat Methods* **14**, 290-296 (2017).
3. Punjani, A. & Fleet, D.J. 3D variability analysis: Resolving continuous flexibility and discrete heterogeneity from single particle cryo-EM. *J Struct Biol* **213**, 107702 (2021).
4. Ma, H. et al. Auto-DRRAFTER: Automated RNA Modeling Based on Cryo-EM Density. *Methods Mol Biol* **2568**, 193-211 (2023).
5. Kappel, K. et al. Accelerated cryo-EM-guided determination of three-dimensional RNA-only structures. *Nat Methods* **17**, 699-707 (2020).
6. Ali, S.E., Mittal, A. & Mathews, D.H. RNA Secondary Structure Analysis Using RNAstructure. *Curr Protoc* **3**, e846 (2023).
7. Liebschner, D. et al. Macromolecular structure determination using X-rays, neutrons and electrons: recent developments in Phenix. *Acta Crystallogr D Struct Biol* **75**, 861-877 (2019).
8. Williams, C.J. et al. MolProbity: More and better reference data for improved all-atom structure validation. *Protein Sci* **27**, 293-315 (2018).
9. Pettersen, E.F. et al. UCSF Chimera--a visualization system for exploratory research and analysis. *J Comput Chem* **25**, 1605-12 (2004).
10. Rigsby, R.E. & Parker, A.B. Using the PyMOL application to reinforce visual understanding of protein structure. *Biochem Mol Biol Educ* **44**, 433-7 (2016).
