# Supplementary material for: The p53 mRNA exhibits riboswitch-like features under DNA damage conditions
Source: iScience. 2025 Sep 12;28(10):113555. doi: 10.1016/j.isci.2025.113555 (PMC12510224; doi:10.1016/j.isci.2025.113555)
Supplement: Data S1. Supporting data sets [file mmc3.pdf]

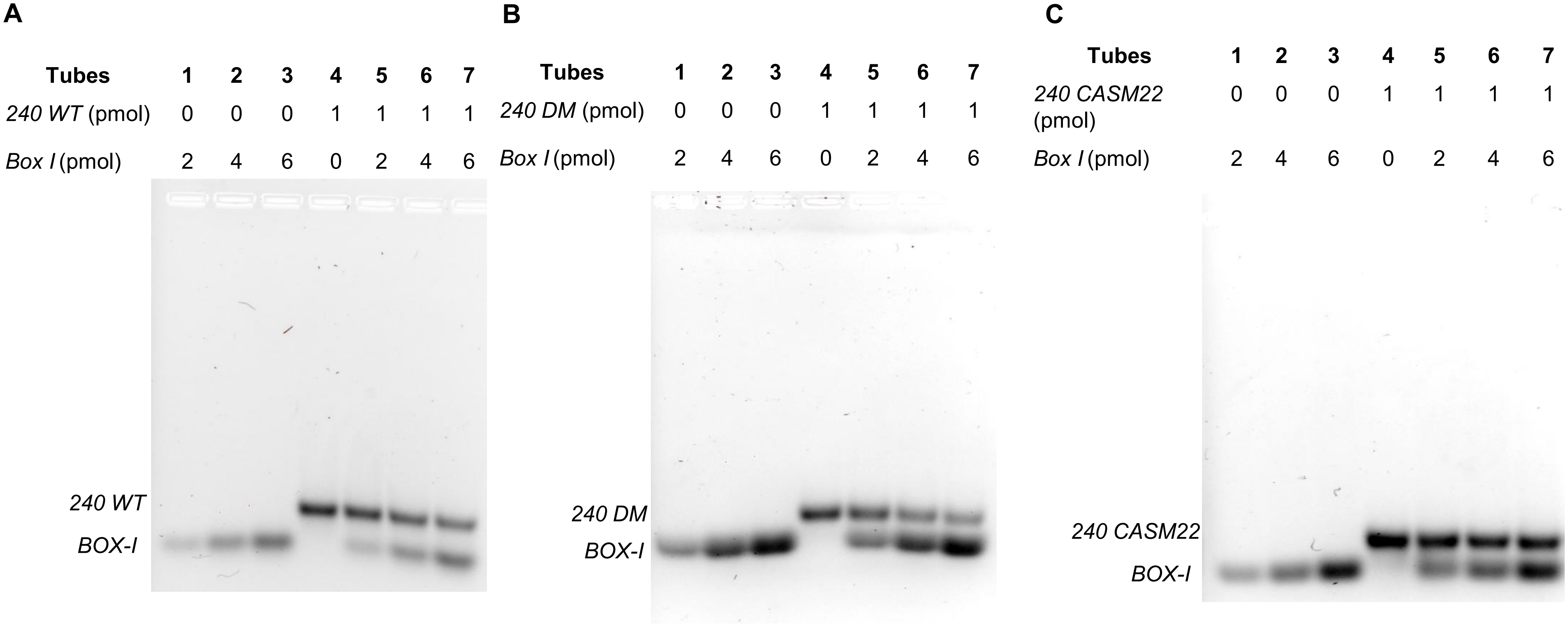

p53 240 WT RNA (A), *p53 240 DM* RNA (B), *p53 240 CASM22* RNA (C), and *BOX-I* oligonucleotide gel shifts.

RNA-SHAPE-MaP profile p53-WT

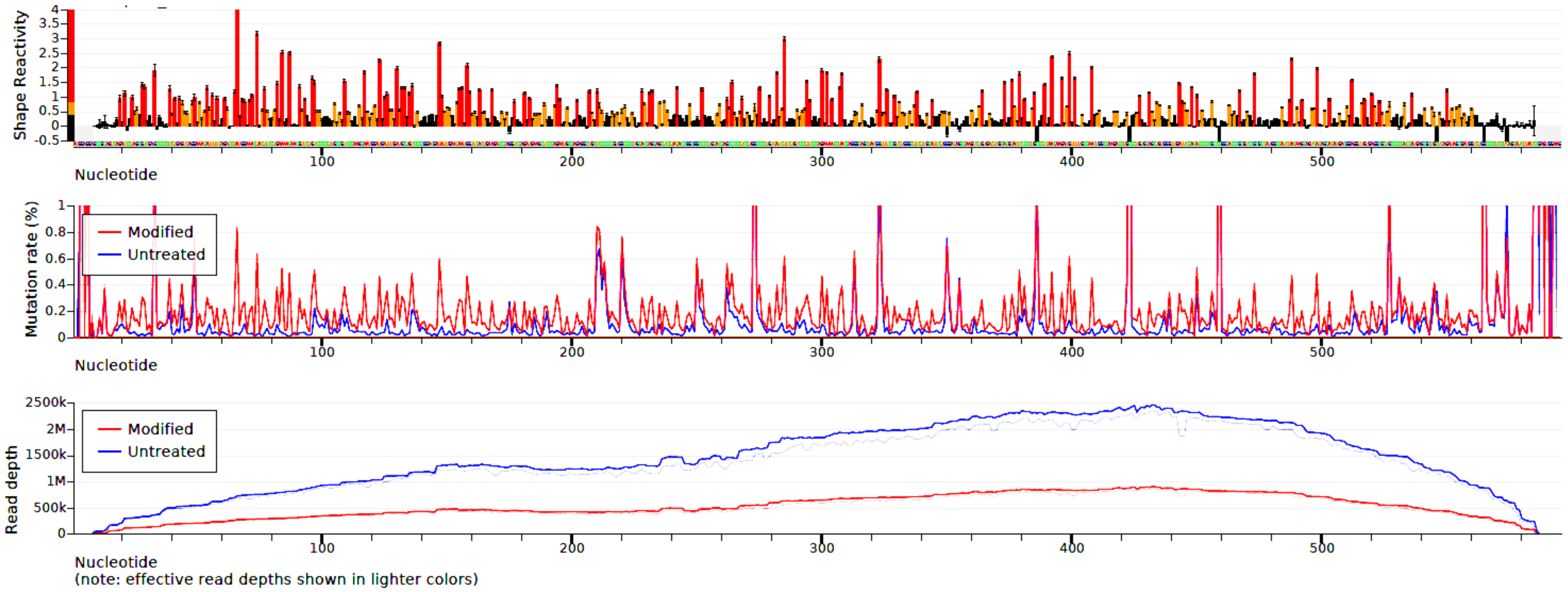

RNA-SHAPE-MaP profiles of *p53-WT* mRNA. The upper panel shows the SHAPE reactivity profiles, higher reactivities are indicated by red bars; the middle panel shows the mutation rate; and the lower panel shows the sequence read depth. RNA modified with 1M7 (modified) is indicated in red, and RNA treated with DMSO control (untreated) is indicated in blue.

RNA-SHAPE-MaP profile p53-CASM22

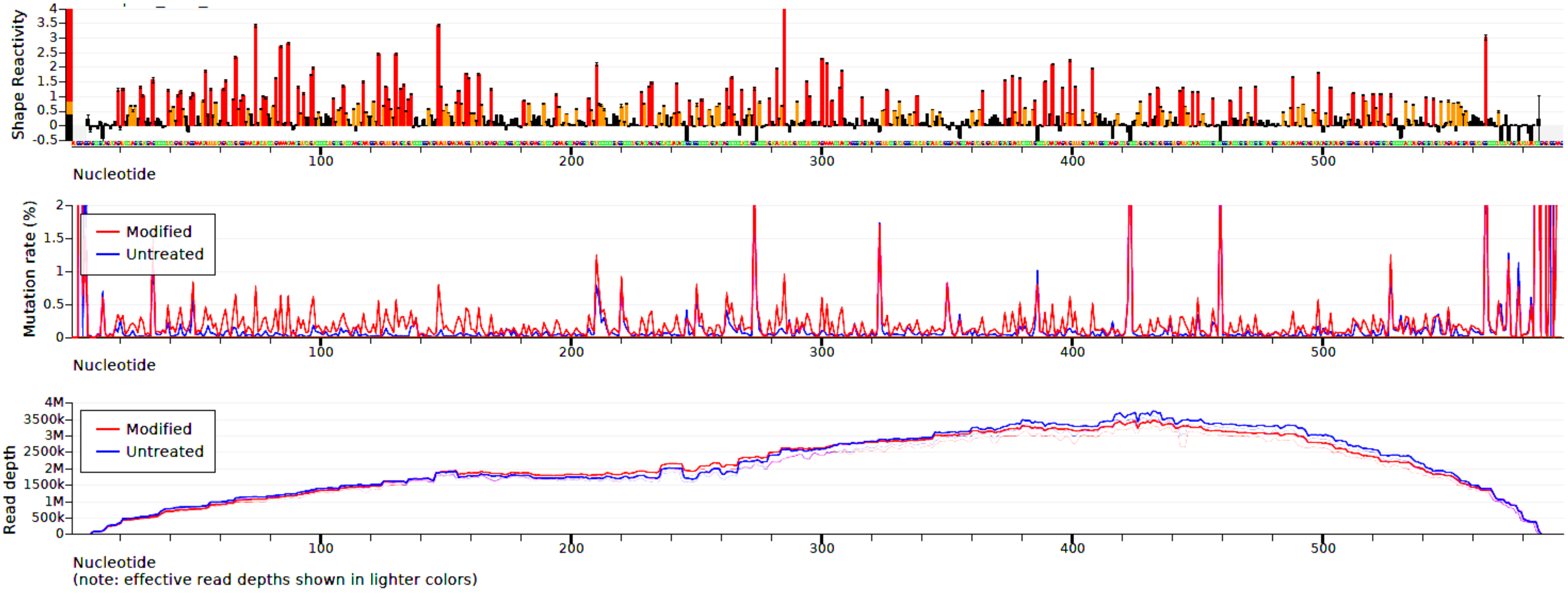

RNA-SHAPE-MaP profiles of *p53-CASM22* mRNA. The upper panel shows the SHAPE reactivity profiles, higher reactivities are indicated by red bars; the middle panel shows the mutation rate; and the lower panel shows the sequence read depth**.** RNA modified with 1M7 (modified) is indicated in red, and RNA treated with DMSO control (untreated) is indicated in blue.

RNA-SHAPE-MaP profile p53-DOXO

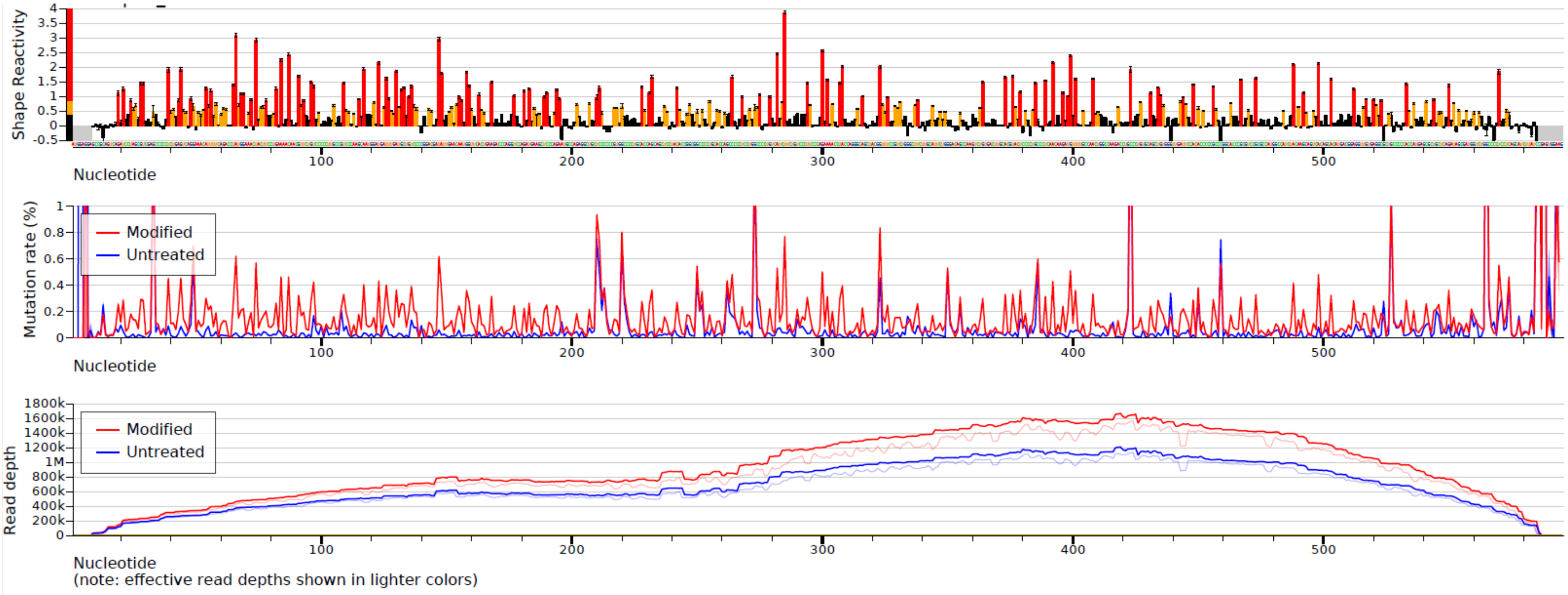

RNA-SHAPE-MaP profile of *p53* mRNA with doxorubicin treatment. The upper panel shows the SHAPE reactivity profiles, higher reactivities are indicated by red bars; the middle panel shows the mutation rate; and the lower panel shows the sequence read depth**.** RNA modified with 1M7 (modified) is indicated in red, and RNA treated with DMSO control (untreated) is indicated in blue.

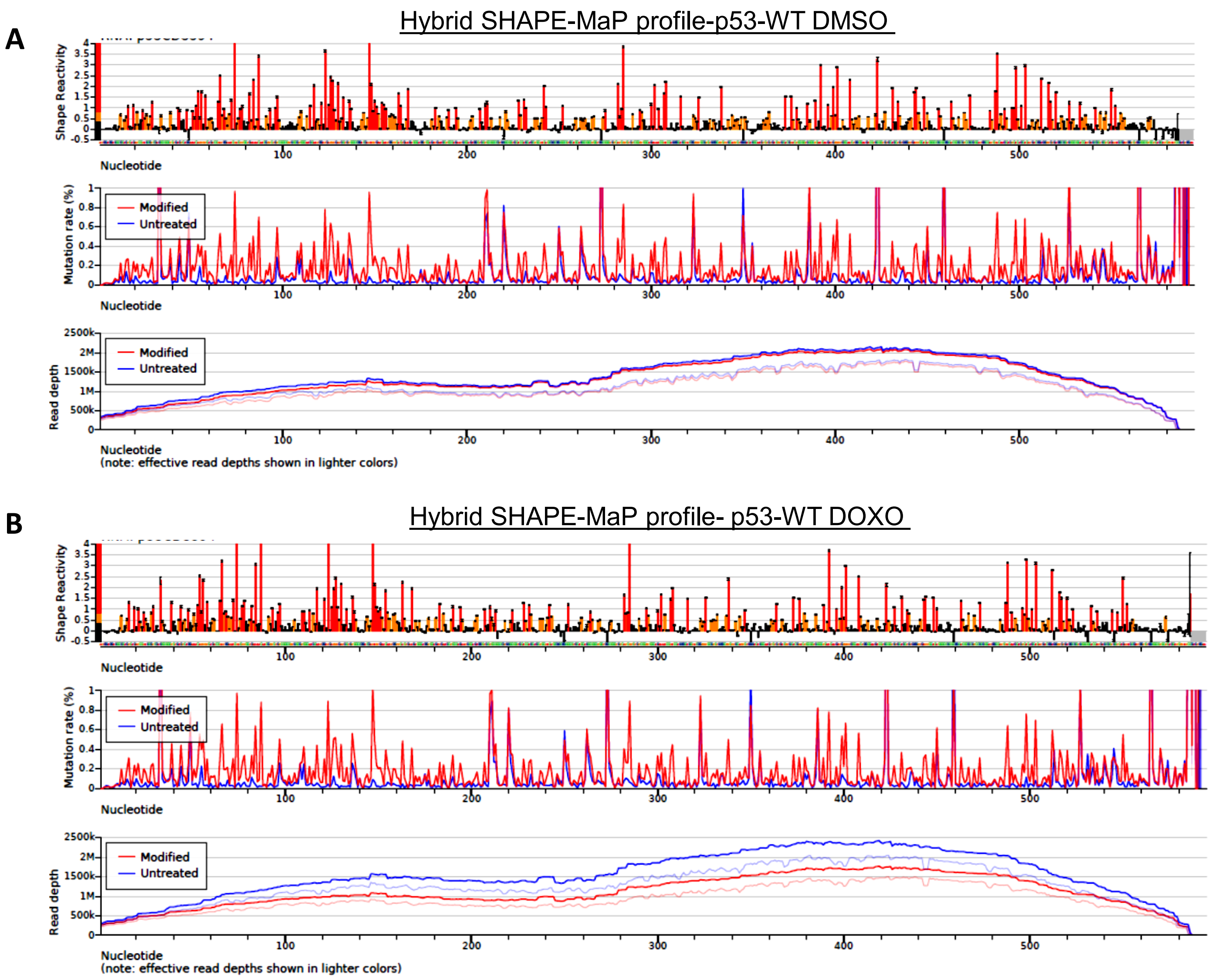

Hybrid SHAPE-MaP profiles (representative) of *p53-WT* mRNA with DMSO (**A**) and DOXO (**B**) treatment. The upper panel shows the SHAPE reactivity profiles, higher reactivities are indicated by red bars; the middle panel shows the mutation rate; and the lower panel shows the sequence read depth**.** RNA modified with 1M7 (modified) is indicated in red, and RNA treated with DMSO control (untreated) is indicated in blue.

In vitro SHAPE-MaP profile- *p53-WT* mRNA

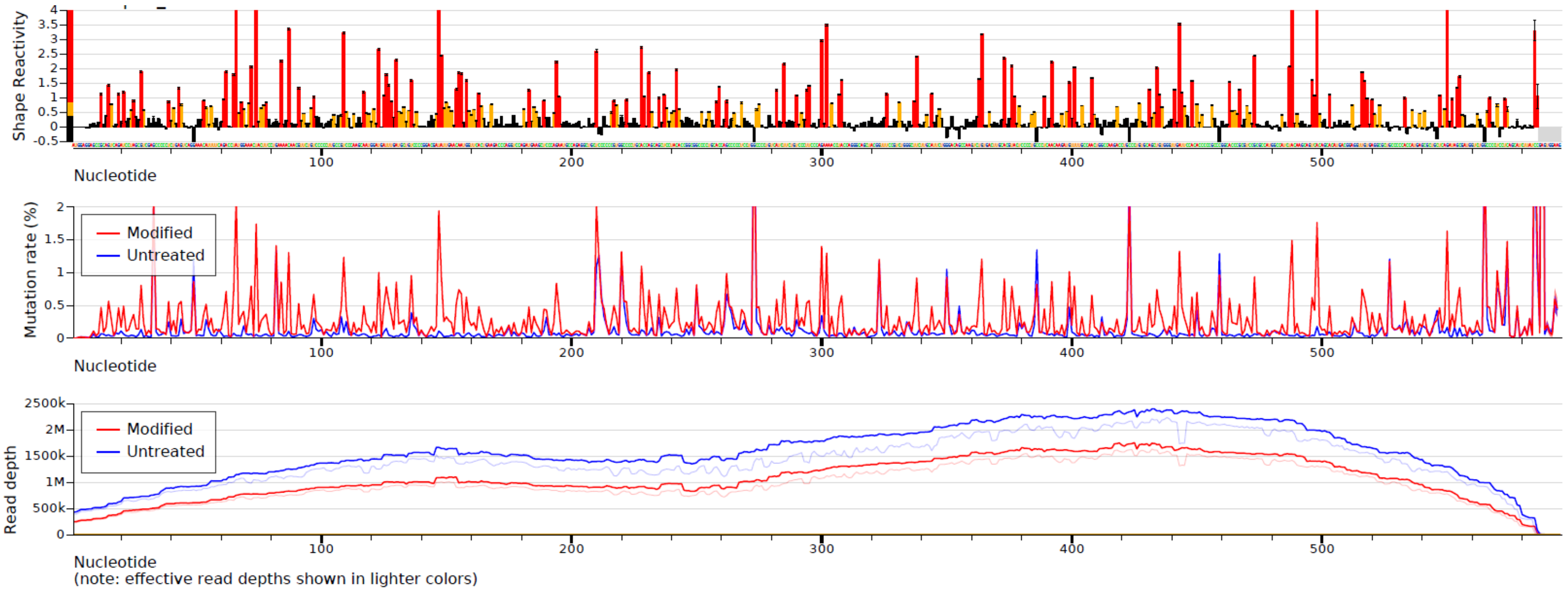

In vitro RNA-SHAPE-MaP profile of *p53-WT* mRNA. The upper panel shows the SHAPE reactivity profiles, higher reactivities are indicated by red bars; the middle panel shows the mutation rate; and the lower panel shows the sequence read depth. RNA modified with 1M7 (modified) is indicated in red, and RNA treated with DMSO control (untreated) is indicated in blue.

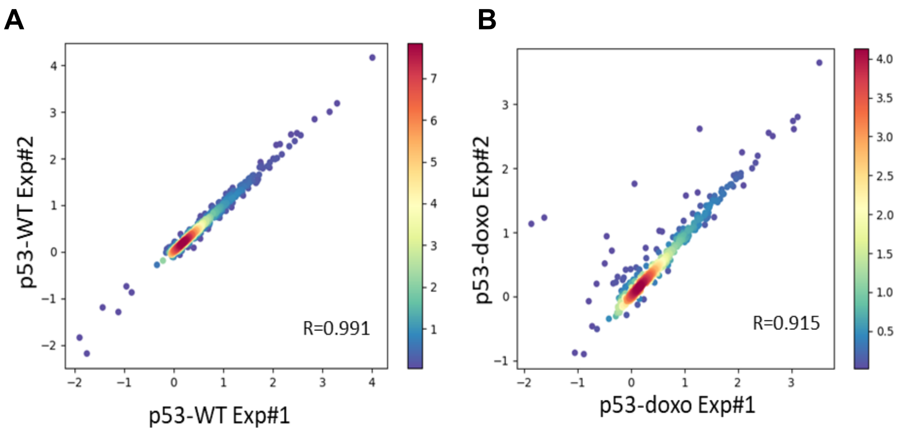

Correlation analysis of RNA SHAPE-MaP from representative independent replicates of the indicated experiments. A) p53-WT; B) p53-doxo. The biological replicates of each set of SHAPE-MaP experiments show excellent greement with Spearman R value >0.9

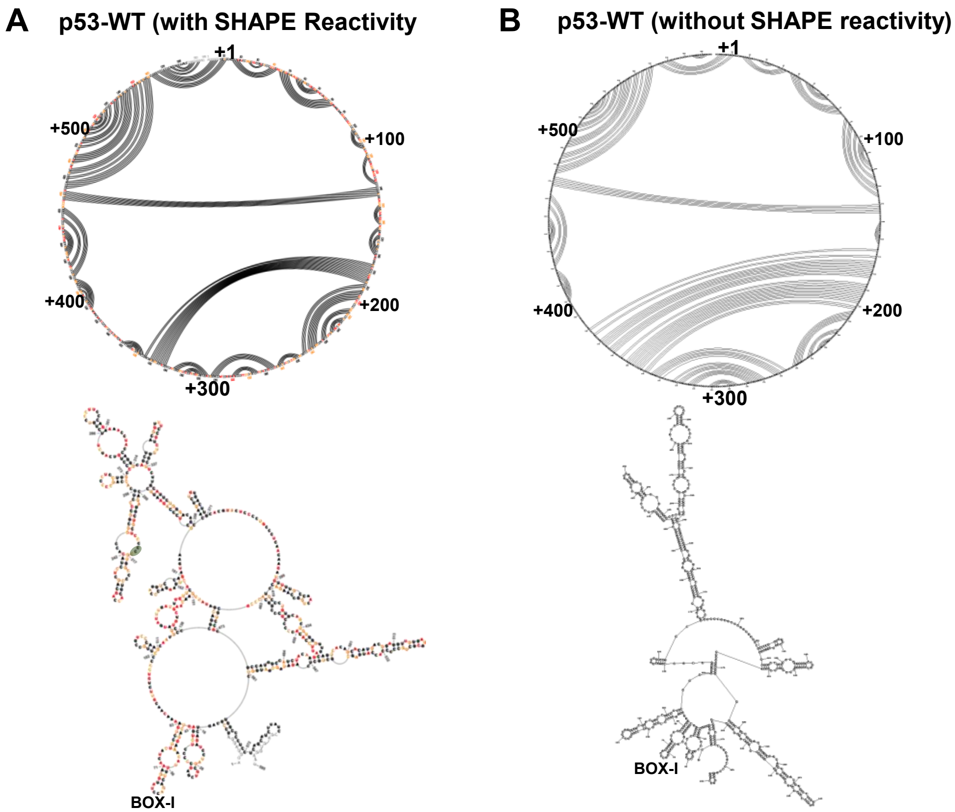

Secondary structure of p53 WT RNA modelled using the SuperFold, with **(A)** and without **(B)** SHAPE reactivity constraint.
